# State of the nation: Understanding the current NHS treatment pathway to identify opportunities to advance future care of patients with high‐risk non‐muscle invasive bladder cancer in the UK (SPAN‐UK)

**DOI:** 10.1002/bco2.70236

**Published:** 2026-06-28

**Authors:** Jonathan Aning, James W. F. Catto, Rebecca Martin, Kathryn Chatterton, Paramananthan Mariappan, Edward Ottley, Joseph Hickey, Stephen McCormack, Simran Gill, Bernadett Szabados

**Affiliations:** ^1^ Bristol Urological Institute North Bristol NHS Trust Bristol UK; ^2^ Population Health Sciences, Bristol Medical School University of Bristol Bristol UK; ^3^ Division of Clinical Medicine, School of Medicine and Population Health University of Sheffield Sheffield UK; ^4^ The Royal Marsden NHS Foundation Trust London UK; ^5^ Guy's and St Thomas' NHS Foundation Trust London UK; ^6^ Edinburgh Bladder Cancer Surgery (EBCS), Western General Hospital University of Edinburgh Edinburgh UK; ^7^ Real World Evidence OPEN Health Communications LLP London UK; ^8^ J&J Innovative Medicine High Wycombe UK; ^9^ University College London Hospitals NHS Foundation Trust London UK

**Keywords:** BCG, bladder cancer, cross‐sectional survey, HR‐NMIBC, non‐muscle invasive bladder cancer, QPI, radical cystectomy, real‐world, treatment pathway

## Abstract

**Objectives:**

This study aimed to understand clinical pathways for patients with high‐risk non‐muscle‐invasive bladder cancer (HR‐NMIBC) from diagnosis to follow‐up and to identify opportunities to improve care.

**Materials and Methods:**

A cross‐sectional survey was conducted via structured online interviews with consented NHS healthcare professionals (HCPs) from the United Kingdom (UK) between June and September 2025. Topics surveyed included MDT structures/roles, diagnostic timelines, adjuvant treatment, radical cystectomy (RC) decision making, current bladder‐sparing treatment and clinical trial access. Quantitative data were analysed descriptively. Qualitative responses were analysed thematically.

**Results:**

Seventy HCPs were included and reported that typically; 88.5% of patients achieve diagnosis within 6–8 weeks of referral, and 11.4% reported delays beyond 8 weeks. BCG maintenance duration and completion rates varied. Following BCG induction, a median (IQR) of 20.0% (5.0–32.5%) and 60.0% (40.0–70.0%) of patients completed ≥2 or ≤1 years of maintenance, respectively; 1.0% (1.0–2.0%) failed to complete induction. For BCG‐unresponsive HR‐NMIBC, HCPs reported that a mean (*SD*) proportion of 53.4 (18.1)% of patients tend to be eligible for and consent to RC, 22.0 (12.6)% tend to be eligible but decline RC and 24.6 (14.9)% tend to be ineligible. Bladder‐sparing options remain limited, with 60% of HCPs regarding further BCG as the most appropriate option. All respondents agreed that adherence to quality performance indicators (QPIs) and a national bladder cancer audit would be beneficial. Insufficient specialist nurse capacity to meet foreseeable demands of HR‐NMIBC patient care was reported by 70% (*n* = 49) of HCPs.

**Conclusion:**

Results reveal variability in real‐world HR‐NMIBC care within the NHS. Delays in diagnosis, inconsistent BCG maintenance duration, lack of evidence‐based alternatives to BCG and a lack of bladder‐sparing treatment and trial options in the BCG‐unresponsive setting were identified. Findings highlight unmet needs in relation to MDT resourcing, diagnostic efficiency, trial access, QPI adherence and a national bladder cancer audit.

## 1 INTRODUCTION

Bladder cancer continues to pose a substantial health challenge in the United Kingdom (UK), with approximately 10 500–18 000 new cases diagnosed annually.^1^, ^2^ Most of these cases (~70%) are classified as non‐muscle‐invasive bladder cancer (NMIBC) at diagnosis.^2^, ^3^, ^4^ NMIBC encompasses a heterogeneous group of tumours stratified into low, intermediate, high‐risk and very high‐risk categories, with high‐risk NMIBC (HR‐NMIBC) representing a subgroup at higher risk for recurrence and progression to muscle‐invasive disease.^5^ Accurate risk stratification is therefore essential to guide surveillance intensity and therapeutic interventions, aiming to balance oncological control with quality of life.^6^

The standard of care (SOC) for HR‐NMIBC in the UK is transurethral resection of bladder tumour (TURBT) followed by intravesical Bacillus Calmette–Guérin (BCG) immunotherapy, with maintenance therapy recommended for up to 3 years.^6^, ^7^, ^8^ Primary radical cystectomy (RC) remains a definitive option that can confer durable oncological control in appropriately selected patients, albeit balanced against possible perioperative morbidity and long‐term quality‐of‐life implications.^9^, ^10^ Despite established guidelines, real‐world practice can be challenged by variability in diagnostic timelines, BCG maintenance duration, and access to specialist multidisciplinary team (MDT) input.^6^ Furthermore, there is a persistent lack of evidence‐based, bladder‐sparing treatment options for BCG‐unresponsive patients who are ineligible for or decline RC, when patients cannot tolerate or progress on BCG, leaving a substantial proportion of HR‐NMIBC patients with limited options.^11^, ^12^

Guidance for the management of HR‐NMIBC in the UK is primarily informed by the National Institute for Health and Care Excellence (NICE) guidelines, which were last updated in 2015.^8^ However, advances in clinical practice and emerging evidence have outpaced these recommendations, resulting in a reliance on other guidance such as European Association of Urology (EAU) guidelines, International Bladder Cancer Group (IBCG) consensus guidelines or local protocols to inform decision‐making.^6^, ^13^ This may contribute to inter‐regional variability in care across the UK in terms of diagnostic efficiency, time to definitive treatment, BCG administration and maintenance duration. Optimising the structure and resourcing of MDTs is also a critical consideration to ensure capacity to meet the demands of HR‐NMIBC care in the real‐world clinical environment.^5^

To date, no single clinician‐informed, national assessment has comprehensively mapped the HR‐NMIBC care pathway across all National Health Service (NHS) regions. Previous studies and reports by the British Association of Urological Surgeons (BAUS) and National Institute for Health and Care Excellence (NICE) have assessed elements of HR‐NMIBC care and have highlighted delays to RC and the impacts of BCG shortages.^14^, ^15^, ^16^, ^17^, ^18^, ^19^ However, these studies and reports are fragmented and do not provide a comprehensive snapshot of the complete treatment pathway from diagnosis to follow‐up.^17^, ^18^, ^19^ To meet this evidence gap, this descriptive, cross‐sectional study was undertaken to characterise current patient management and treatment pathways from diagnosis through to follow‐up to identify areas for optimisation and understand the evolving MDT structure and decision‐making processes. With the aim of informing future research, support the development of updated clinical guidelines, and drive pathway optimisation to improve outcomes for HR‐NMIBC patients across the UK.

## 2 MATERIALS AND METHODS

### 2.1 Study design and participants

This cross‐sectional study utilised a survey‐based methodology, which was developed with expert input from a steering group of UK clinical experts to ensure clinical relevance. Between June and September 2025, structured online interviews were conducted with 70 consented NHS HCPs who expressed interest in participating after being approached by the study sponsor's medical team. Participants were routinely involved in the care of patients with HR‐NMIBC, and interviews were supported by the senior medical science liaison (MSL) from the sponsor's medical team. The survey platform captured both quantitative and qualitative data, providing national representation across institutions and roles. Respondents either consulted locally available databases to inform their responses or provided estimates based on their professional experience where databases were incomplete or unavailable.

### 2.2 Objectives and outcomes

The primary objectives of this study were to map HR‐NMIBC management pathways from diagnosis through to follow‐up, to identify opportunities for care optimisation and to describe MDT structure and decision‐making processes. Secondary objectives were to assess diagnostic timelines and BCG maintenance duration, to evaluate bladder‐sparing treatments and clinical trials for RC‐ineligible or declining patients and to document unmet needs and resource constraints.

### 2.3 Statistical methods

Quantitative data were analysed descriptively, with frequencies, means, medians and interquartile ranges (IQRs) calculated for categorical and continuous variables. No inferential statistics or hypothesis testing was performed, consistent with the descriptive nature of the study. Qualitative free‐text responses underwent thematic analysis using an inductive approach, involving systematic coding of each response in turn to identify themes that represent a narrative of real‐world challenges. Data were synthesised to highlight regional differences in diagnostic timelines, BCG maintenance protocols and access to bladder‐sparing treatment options and clinical trials. A formal sample size calculation was not performed, as this cross‐sectional study aimed to provide a comprehensive national snapshot rather than test specific hypotheses. The target sample of 70 HCPs was determined pragmatically to ensure broad representation across NHS regions and professional roles.

### 2.4 Ethical considerations

All data were anonymised and securely stored in compliance with the General Data Protection Regulation (GDPR) and the Declaration of Helsinki. Written informed consent was obtained from each participant prior to interview. As the study involved NHS staff voluntarily providing professional opinions rather than individual patient‐level data, formal ethics approval was not required. Participants' rights and confidentiality were protected throughout.

## 3 RESULTS

### 3.1 HCPs characteristics

HCP characteristics are summarised in Table 1. A total of 70 NHS HCPs were included, representing 20 Cancer Alliances across England (87.1%), Scotland (4.3%), Wales (4.3%) and Northern Ireland (4.3%). Of the respondents, 32.9% (*n* = 23) worked in district general hospitals; most HCPs (64.3%, *n* = 45) were specialist urology doctors at consultant, staff grade/middle grade and surgical fellow level. A total of 27.1% (*n* = 19) specialist nurse team members completed the survey (including clinical nurse specialists, advanced nurse practitioners and other specialist nurses); the remaining 8.6% (*n* = 6) were clinical oncologists.

**TABLE 1:** Characteristics of HCP participants.

	Number of respondents	%
Respondent role (*N* = 70)		
Advanced nurse practitioner (ANP)/dedicated bladder cancer clinical nurse specialist (CNS)	4	5.7
Urology/uro‐oncology specialist nurse	10	14.3
Consultant urologist	40	57.1
Staff Grade urologist/surgical fellow	5	7.1
Clinical oncologist	6	8.6
Other^a^	5	7.1
Location of respondents (*N* = 70)		
England^b^	61	87.1
Northern Ireland^c^	3	4.3
Scotland^d^	3	4.3
Wales^e^	3	4.3
Main practice setting (*N* = 70)		
District general hospital	23	32.9
University teaching hospital or specialist cancer centre	47	67.1
How many NMIBC patients overall are typically discussed per month at MDT meetings?		
<10	0	0.0
10–15	5	7.1
15–20	9	12.9
20–25	27	38.6
25–30	14	20.0
>30	15	21.4
Of these, NMIBC cases, what proportion tend to present with CIS (carcinoma in situ)?		
<10%	25	35.7
≥10% ≤ 15%	29	41.4
>15%	6	8.6
Do not know	10	14.3
Proportion of new bladder cancer patients diagnosed with NMIBC at the HCP's site, mean (*SD*) %	72.5 (6.1)	
Proportion of new bladder cancer patients diagnosed with muscle‐invasive disease at the HCP's site, mean (*SD*) %	27.4 (6.2)	

^a^ Other responses: nurse consultant within urology (*n* = 1); urology oncology lead nurse (*n* = 1); urology nurse practitioner (*n* = 1); specialist urology diagnostic nurse (*n* = 1); urology nurse practitioner (bladder cancer team) (*n* = 1).

^b^ Regional cancer alliances: London (North Central London Cancer Alliance [*n* = 1]; North East London Cancer Alliance [*n* = 1]; RM Partners [*n* = 5]; South East London Cancer Alliance [*n* = 3]); South East (Kent and Medway Cancer Alliance [*n* = 1]; Surrey and Sussex Cancer Alliance [*n* = 4]); South West (Peninsula Cancer Alliance [*n* = 4]; Somerset, Wiltshire, Avon and Gloucestershire Cancer Alliance [*n* = 2]; Thames Valley Cancer Alliance [*n* = 5]; Wessex Cancer Alliance [*n* = 2]); West Midlands (West Midlands Cancer Alliance [*n* = 3]); East Midlands (East Midlands Cancer Alliance [*n* = 5]); North East (Northern Cancer Alliance [*n* = 3]; South Yorkshire and Bassetlaw Cancer Alliance [*n* = 7]); North West (Lancashire and South Cumbria Cancer Alliance [*n* = 5]; Cheshire and Merseyside Cancer Alliance [*n* = 6]; Greater Manchester Cancer Alliance [*n* = 4]).

^c^ Regional cancer alliance: NHS Northern Ireland (*n* = 3).

^d^ Regional cancer alliance: NHS Scotland (*n* = 3).

^e^ Regional cancer alliance: NHS Wales (*n* = 3).

HCP respondents reported that, on average, 72.5% (standard deviation [*SD*] 6.1%) of new bladder cancer diagnoses at their centres were NMIBC, and 27.4% (*SD* 6.2%) were muscle‐invasive. The number of NMIBC cases discussed monthly at MDTs varied: 20.0% (*n* = 14) typically discussed 10–20 NMIBC cases per month, 58.6% (*n* = 41) discussed 20–30 and 21.4% (*n* = 15) discussed more than 30 NMIBC cases per month. The reported proportion of NMIBC cases discussed at MDTs presenting with carcinoma in situ (CIS) disease varied as follows: 35.7% (*n* = 25) indicated that CIS accounted for less than 10% of NMIBC cases; 41.4% (*n* = 29) reported a proportion between 10% and 15%; and 8.6% (*n* = 6) reported that more than 15% of NMIBC cases presented with CIS.

### 3.2 MDT team structure and leadership

Most HCPs (62.9%, *n* = 44) reported that their centre operated a local uro‐oncology MDT including a bladder cancer section, whereas 21.4% (*n* = 15) functioned as super‐regional specialist MDTs. Only 7.1% (*n* = 5) reported dedicated bladder cancer MDTs, and 8.6% (*n* = 6) described hybrid models involving both local and regional uro‐oncology MDTs (Table 2). Across all sites, consultant urologists led key decisions regarding TURBT, risk stratification and adjuvant therapy. Specialist nurse team members played a central role in intravesical therapy instillation (95.7%, *n* = 67), holistic needs assessments (88.6%, *n* = 62) and psychological support (82.9%, *n* = 58) (all roles and responsibilities are summarised in Tables S1 and S2). A total of 70.0% (*n* = 49) of the respondents reported insufficient specialist nurse resourcing to meet foreseeable demands of HR‐NMIBC patient care.

**TABLE 2:** MDT structure.

	Number of respondents	%
Local MDT where HR‐NMIBC patients are discussed (*N* = 70)		
Have a local uro‐oncology MDT (with bladder cancer section)	44	62.9
Have a dedicated local MDT that only discusses bladder cancer patients	5	7.1
Act as the super‐regional specialist bladder cancer MDT	15	21.4
Other^a^	6	8.6

^a^ Other responses: ‘It is not super‐regional but is part of North West region only where the MDT covers 3 different trusts (level 2 specialist MDT)’ (*n* = 1); ‘both super‐regional bladder cancer MDT and a local uro‐oncology MDT (with bladder section)’ (*n* = 1); ‘2 local uro‐oncology MDTs (with bladder section) and 1 network MDT’ (*n* = 1); ‘local and regional uro‐oncology MDT with bladder section’ (*n* = 1); ‘they do have a local uro‐oncology MDT but also act as a regional MDT both have a bladder section’ (*n* = 1); ‘4 local and 1 regional network MDT’ (*n* = 1).

### 3.3 Diagnostic pathway and timelines

Reported diagnostic timelines, guideline use and diagnostic tools are summarised in Table 3. New bladder cancer referrals predominantly arise via General Practitioner (GP) to haematuria or 2‐week wait (2WW) clinics (88.6%, *n* = 62) in the NHS. Most HCPs (84.3%, *n* = 59) found the diagnosing and staging aspects of the 2015 NG2 bladder cancer diagnosis and management guidelines most relevant to current routine management, with 70.0% finding these guidelines useful for follow‐up after treatment. A total of 92.9% (*n* = 65) reported finding EAU guidelines most useful for current management; only 1.4% (*n* = 1) of HCPs found the Getting It Right First Time (GIRFT) guidelines most useful for current management. In terms of diagnostic timelines, a total of 1.4% (*n* = 1/70) of HCPs reported that an initial urologist appointment is held within 1 week of referral, 45.7% (*n* = 32) reported that this is held within 2 weeks of referral and 52.9% (*n* = 37) reported that this occurs beyond 2 weeks. Diagnostic flexible cystoscopy was typically performed within 2 weeks for 72.9% (*n* = 51), with 5.7% (*n* = 4/70) reporting that this occurs within 1 week. For TURBT, 8.6% (*n* = 6/70) reported that this occurs within 2 weeks and 40.0% (*n* = 28) of HCPs reported that TURBT occurs within 3 weeks; 51.4% (*n* = 36) reported this taking beyond 3 weeks. Pathology review and staging were reported to be completed within 1 week by 4.3% (*n* = 3/70) of HCPs and within 2 weeks for 72.9% (*n* = 51) of HCPs. Diagnosis was reported to be communicated with patients within 1 week of pathology by 65.7% (*n* = 46) of HCPs. Overall, 1.4% (*n* = 1/70) reported that a confirmed diagnosis was received up to 4 weeks from referral, 40% (*n* = 28) up to 6 weeks, 47.1% (*n* = 33) within 8 weeks and 11.4% (*n* = 8) of HCPs reported delays exceeding 8 weeks.

**TABLE 3:** The time taken for activities associated with diagnosis according to the experience of HCPs.

Activity/typical time taken/referral/guidelines used/diagnostic tools	Number of respondents (*N* = 70)	%
Time taken for an initial appointment with a urologist		
Within 1 week	1	1.4
Within 2 weeks	32	45.7
Over 2 weeks	37	52.9
Time taken for an initial diagnostic flexible cystoscopy		
Within 1 week	4	5.7
Within 2 weeks	51	72.9
Within 3 weeks	11	15.7
Over 3 weeks	4	5.7
Time taken for TURBT		
Within 1 week	0	0.0
Within 2 weeks	6	8.6
Within 3 weeks	28	40.0
Over 3 weeks	36	51.4
Time taken for pathology review/staging		
Within 1 week	3	4.3
Within 2 weeks	51	72.9
Over 2 weeks	16	22.9
Time taken for diagnosis communication with patients		
Within 1 week of pathology report	46	65.7
Within 2 weeks of pathology report availability	24	34.3
Overall typical timeframe from referral to a confirmed and communicated diagnosis of HR‐NMIBC		
Up to 4 weeks	1	1.4
Up to 6 weeks	28	40.0
Up to 8 weeks	33	47.1
More than 8 weeks	8	11.4
Tools most routinely utilised by the respondent's team at the time of diagnosis		
White light cystoscopy	70	100.0
Photodynamic diagnosis (PDD)/blue light cystoscopy	25	35.7
Narrow‐band imaging cystoscopy (NBI)	29	41.4
Urine cytology	37	52.9
CT urography	70	100.0
PET‐CT	8	11.4
MRI	27	38.6
Ultrasound	48	68.6
Other^a^	1	1.4
Aspects of the 2015 NICE (NG2) bladder cancer diagnosis and management guidelines most relevant to current routine management		
Information and support for people with bladder cancer	29	41.4
Diagnosing and staging bladder cancer	59	84.3
Treating NMIBC	7	10.0
Follow‐up after treatment for NMIBC	49	70.0
Treating MIBC	3	4.3
Follow‐up after treatment for MIBC	25	35.7
Managing locally advanced or metastatic urothelial carcinoma	2	2.9
Specialist palliative care for people with incurable bladder cancer	20	28.6
I do not follow any of the current NICE bladder cancer guidelines (2015)	8	11.4
Guidelines/recommendations found most useful to inform current management of HR‐NMIBC		
NICE (National Institute for Health and Care Excellence) Bladder cancer: diagnosis and management guidelines (2015)	2	2.9
EAU guidelines (European Association of Urology)	65	92.9
ESMO guidelines (European Society for Medical Oncology)	0	0.0
GIRFT (Getting it Right First Time) Urology Guidance for Bladder Cancer	1	1.4
AUA guidelines (American Urological Association)	0	0.0
EORTC guidelines (European Organisation for Research and Treatment of Cancer)	0	0.0
IBCG guidelines (International Bladder Cancer Group)	0	0.0
NCCN® guidelines (National Comprehensive Cancer Network®)	0	0.0
Local/regional guidelines	2	2.9
Other	0	0.0

^a^ Other: ‘NBI is used during flexible cystoscopy, whereas white/blue light is at TURBT. More typically white light is used. Blue light is reserved for cases considered to be of higher risk’ (*n* = 1).

### 3.4 Risk stratification

Risk stratification was assessed in line with the risk groups highlighted in EAU guidelines.^20^ In terms of risk stratification, HCPs estimated a mean (*SD*) proportion of 33.2 (13.1)% of patients to be low‐risk, 26.8 (9.2)% to be intermediate risk and 40.0 (11.2)% to be high risk (Table 4).

**TABLE 4:** Risk stratification and patient characteristics according to the experience of HCPs.

Proportion of NMIBC patients risk stratified into low‐, intermediate‐ and high‐risk subgroups, median (IQR) %	*N* = 70
Low risk	32.0 (25.0–40.)
Intermediate risk	25.0 (20–32.3)
High risk	40.0 (30.0–50.0)
No risk stratification recorded	0.0 (0.0–0.0)

### 3.5 BCG completion and real‐world challenges

HCPs reported that following BCG induction, a median (IQR) of 20.0% (5.0–32.5%) and 60.0% (40.0–70.0%) of their patients completed ≥2 or ≤1 years of maintenance, respectively, whereas a median (IQR) of 10.0% (10.0–15.0%) only received the minimum level for ‘adequate BCG therapy’ (≥5 doses of BCG induction and ≥2 doses of maintenance as defined by the Food and Drug Administration [FDA] BCG‐unresponsive Treatment Guidance for Industry).^11^ A median (IQR) of 1.0% (1.0–2.0%) failed to complete induction (Table 5).

**TABLE 5:** Intravesical BCG completion rates and RC decisions in patients unresponsive to BCG.

For those HR‐NMIBC patients who receive intravesical BCG what proportion typically complete the following: median (IQR) %	*N* = 67
Complete BCG induction followed by ≥2 years of maintenance	20.0 (5.0–32.5)
Complete BCG induction followed by ≤1 year of maintenance	60.0 (40.0–70.0)
Receive ≥5 doses of BCG induction and ≥2 doses of maintenance	10.0 (10.0–15.0)
Complete at ≥5 doses of BCG induction only	4.0 (3.0–5.0)
Fail to complete BCG induction (<5 doses, due to disease recurrence or unacceptable toxicity/QoL impacts)	1.0 (1.0–2.0)

Proportion of BCG‐unresponsive^a^ high‐grade NMIBC patients that tend to be mean (*SD*) %	*N* = 69
Eligible and consent to RC	53.4 (18.1)
Eligible but decline RC	22.0 (12.6)
Ineligible for RC	24.6 (14.9)

^a^ Persistent/recurrent disease shortly after a full course of BCG, often ≤6 months for T1/Ta high‐grade or ≤12 months for CIS.

**FIGURE 1:**
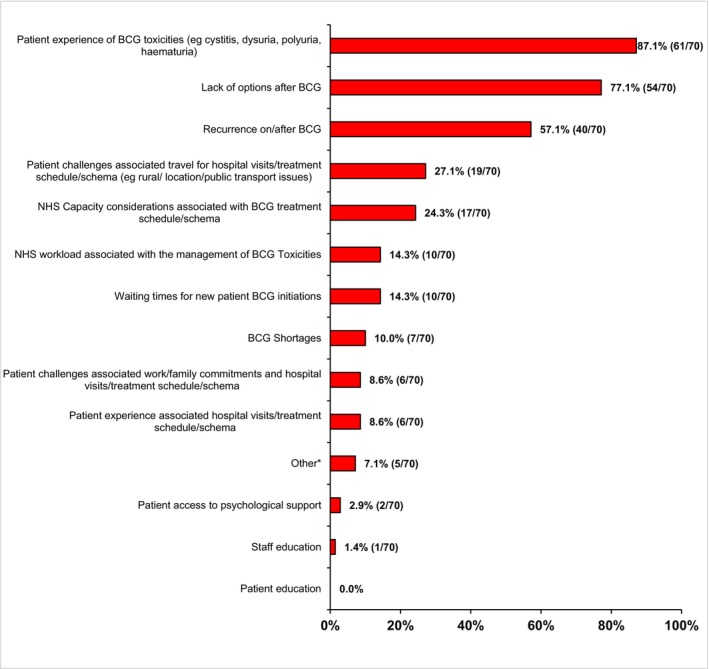
Real‐world challenges associated with BCG use. *Other (summarised): centralisation of resources leading to referral elsewhere for RC, which can lead to BCG re‐induction (*n* = 1); contraindications to BCG (*n* = 1); effective real‐world tracking of HR‐NMIBC on BCG, staff education of International Bladder Cancer Group post‐BCG failure definition (*n* = 1); historic BCG shortages which no longer exist, concerns of future shortages (*n* = 1); patient quality of life is a key overall consideration (*n* = 1).

Frequently reported real‐world challenges associated with BCG instillations included patient experience of toxicities (87.1%, *n* = 61), lack of options after BCG (77.1%, *n* = 54), recurrence on/after BCG (57.1%, *n* = 40), patient challenges associated with hospital visits (27.1%, *n* = 19) and NHS capacity considerations (24.3%, *n* = 17). In addition, NHS workload and waiting times for BCG instillation were reported by 14.3%, and BCG shortages by 10.0% of HCPs (Figure 1).

### 3.6 Radical cystectomy decision‐making

**FIGURE 2:**
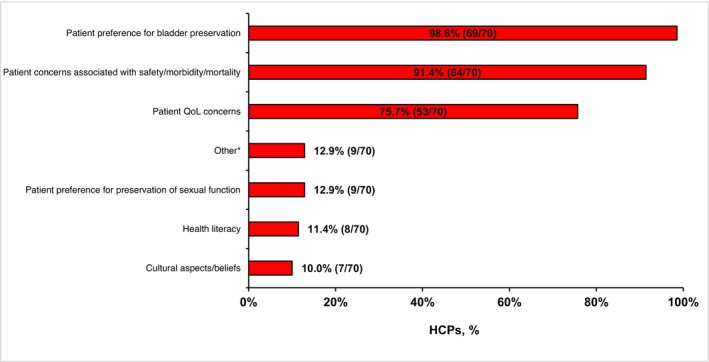
Patient reasons reported by HCPs for not consenting to RC. *Other (summarised): concern about urinary diversion/anxiety about surgery and recovery (*n* = 1); health illiterate patients follow HCP recommendation (*n* = 1); patients are referred specifically in many cases (*n* = 1); patients choosing bladder‐sparing trials (*n* = 1); availability of bladder‐sparing trials with radiotherapy included in standard of care and experimental arms would make more RC eligible patients more confident in choosing bladder‐sparing trial alternative (*n* = 1); open trials or early access programme (*n* = 1); site takes RC referrals so considered by the respondent to be more likely to consent (*n* = 1); in the past 12 months, few (~5%) have refused RC despite being eligible (*n* = 1); quality of life is an important aspect (*n* = 1). Denominator: *n* = 70.

For BCG‐unresponsive NMIBC, as defined in EAU guidelines,^20^ HCPs reported that a mean (*SD*) proportion of 53.4 (18.1)% of patients tend to be eligible for and consent to RC. Whereas a mean (*SD*) proportion of 22.0 (12.6)% of BCG‐unresponsive HR‐NMIBC patients tend to be eligible but decline RC, and 24.6 (14.9)% tend to be ineligible (Table 5). Frequently reported reasons for declining RC related to patient preference for bladder preservation (98.6%), safety/morbidity (91.4%) and quality‐of‐life concerns (75.7%) (Figure 2). Preservation of sexual function (12.9%), health literacy (11.4%) and cultural beliefs (10.0%) also influenced decisions.

### 3.7 Bladder‐sparing and clinical trial options for BCG unresponsive NMIBC

For patients with BCG unresponsive NMIBC, further intravesical BCG was considered the most appropriate by 60.0% (*n* = 42) of HCPs (Table 6). Hyperthermic intravesical chemotherapy (typically either HIVEC® or Synergo®) with MMC was reportedly an option for 27.1% (*n* = 19) of HCPs, but access often required referral to other centres. Clinical trial access was inconsistent across the UK, with 35.7% (*n* = 25) reporting no access to local trial options, 40.0% (*n* = 28) reporting access to trials evaluating alternative intravesical treatments, 27.1% (*n* = 19) accessing trials evaluating immune checkpoint inhibitors and 10.0% (*n* = 7) accessing trials evaluating novel gene, oncolytic therapy or BCG combination trials.

**TABLE 6:** HCP perceptions on appropriate bladder‐sparing treatment and clinical trial options available for patients with BCG unresponsive NMIBC.

For BCG unresponsive NMIBC patients, which of the following bladder sparing treatment options tend to be considered most appropriate?	Number of respondents (*N* = 70)	%
Further intravesical BCG	42	60.0
Intravesical Mitomycin C (MMC)	0	0.0
Hyperthermic Intravesical Chemotherapy (e.g., HIVEC™ or Synergo®) with mitomycin‐C (MMC)	19	27.1
Intravesical Gemcitabine	0	0.0
Sequential Intravesical Gemcitabine & Docetaxel (Gem/Doce)	0	0.0
Sequential BCG and electromotive MMC	0	0.0
Other^a^	9	12.9

At present, for BCG unresponsive NMIBC patients, which (if any) of the following bladder sparing Clinical Trial options can respondent's patients be considered for within the respondent's network?	Number of respondents (*N* = 70)	%
BCG in novel combinations	3	4.3
Immune checkpoint inhibitor monotherapy	7	10.0
Immune checkpoint inhibitor in novel combinations	12	17.1
Novel gene therapy	1	1.4
Selective oncolytic immunotherapy	3	4.3
Alternative Intravesical treatment options	28	40.0
There are no bladder sparing clinical trial options currently recruiting BCG unresponsive NMIBC patients locally	25	35.7
Other^b^	17	24.3

^a^ Other responses: ‘ongoing surveillance with cystoscopy or TULA’ (*n* = 1); ‘radiotherapy with a radiosensitiser’ (*n* = 1); ‘further BCG is offered only if there is a long disease free interval after prior BCG. More commonly hyperthermic intravesical chemotherapy with MMC is considered. Clinical trials are the preferred option for all eligible patients if SOC options like hyperthermic intravesical chemotherapy with MMC are included as comparators’ (*n* = 1); ‘intravesical epirubicin after TURBT in BCG unresponsive NMIBC patients’ (*n* = 1); ‘majority of such cases are considered for trials when possible. If not, then other options are considered’ (*n* = 1); ‘this site utilises SYNERGO Hyperthermic MMC (not HIVEC) (*n* = 1)’; ‘access to hyperthermic MMC using SYNERGO tech (not HIVEC) at a nearby teaching hospital’ (*n* = 1); ‘access to Hyperthermic MMC is available in this trust but the SYNERGO tech is used not HIVEC’ (*n* = 1); ‘bladder sparing trials are open at the moment’ (*n* = 1).

^b^ Other responses: ‘Oncologists have referred a small number of eligible BCG unresponsive NMIBC cases for inclusion in immunotherapy trials’ (*n* = 1); ‘we seek to offer local NHS care options for most cases. Oncologists do refer some BCG‐unresponsive NMIBC patients for immunotherapy trials’ (*n* = 1); ‘patient would have to be referred elsewhere for trials’ (*n* = 1); ‘there are a few bladder‐sparing trial feasibilities underway at present’ (*n* = 1); ‘radiotherapy trial with a radio sensitiser in set up. Hopefully will open in the next 6–12 months’ (*n* = 1); ‘in the bladder sparing setting we may struggle to find patients who meet eligibility for trials and who consent to being referred elsewhere. Many patients in this setting are frail and older age’ (*n* = 1); ‘we have struggled to refer patients in this setting elsewhere for bladder sparing trials due to challenges with eligibility criteria and keeping track of trials’ (*n* = 1); ‘we are setting up a trial which is using HIVEC’ (*n* = 1); ‘HIVEC trial in set up’ (*n* = 1); ‘so there are currently no trials still recruiting at trust level in this setting. However, they do try to keep updated on suitable trials that may be open outside the trust for certain patients’ (*n* = 1); ‘there was a HIVEC based trial at trust level. Patients frequently do not want to go outside of trust for trials where they may struggle with eligibility and travel aspects following years of prior NHS care etc.’ (*n* = 1); ‘radiotherapy as part of trials or associated with local audits case series etc’ (*n* = 1); ‘there are trials in feasibility stage but none yet open and none set to open soon’ (*n* = 1); ‘one of the Urologists is conducting feasibility for a HIVEC trial’ (*n* = 1); ‘GEM/DOCE trial under consideration’ (*n* = 1); ‘HIVEC trial open’ (*n* = 1); ‘there is a HIVEC trial also available locally’ (*n* = 1).

**FIGURE 3:**
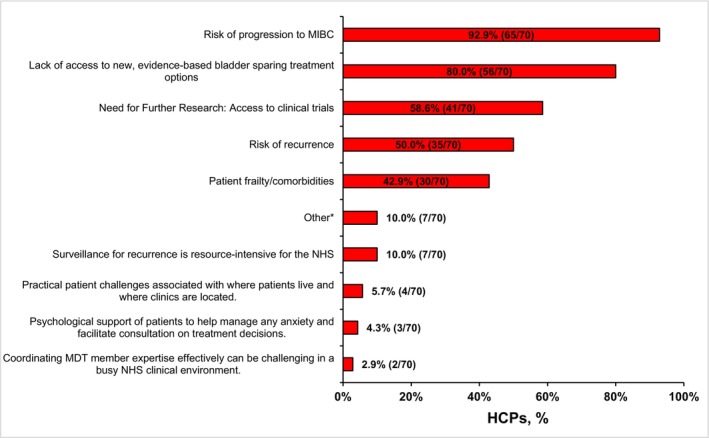
Important HCP concerns when offering bladder‐sparing care to patients with BCG unresponsive NMIBC. *Other (summarised): ‘Pathway delays due to lack of theatre space, clinical capacity and specialist nurse resource are the chief concerns impacting outcomes for this group’ (*n* = 1); ‘for older more heavily pre‐treated patients in the bladder sparing setting. If you don't treat now, then the opportunity to treat later may be lost. Watching and waiting not an option’ (*n*−1); ‘also it is challenging Keeping track of trials that patients can be referred to’ (*n* = 1); ‘lack of CNS resource’ (*n* = 1); ‘pathway delays to TURBT and pathology report availability are the chief concerns impacting outcomes’ (*n* = 1); ‘TULA (Transurethral Laser Ablation) is seen as useful in this setting to debulk patients and reduces the concern of patients presenting at A&E with bleeds associated with bulky disease etc.’ (*n* = 1); ‘the impact of patient choice is an important concern since some want to preserve their bladders despite being eligible for Radical Cystectomy’ (*n* = 1).

Key concerns when offering bladder‐sparing care included risk of progression to muscle‐invasive disease (92.9%, *n* = 65), lack of access to new evidence‐based options (80.0%, *n* = 56), need for further research (58.6%, *n* = 41), risk of recurrence (50.0%, *n* = 35) and patient frailty/comorbidities (42.9%, *n* = 30) (Figure 3).

### 3.8 Quality performance indicators (QPIs) and unmet needs

All respondents agreed that adherence to QPIs could reduce the risk of recurrence and progression while also reducing nationwide variance in NMIBC patient care. In addition, all HCPs reported that a national bladder cancer audit would be beneficial.

**FIGURE 4:**
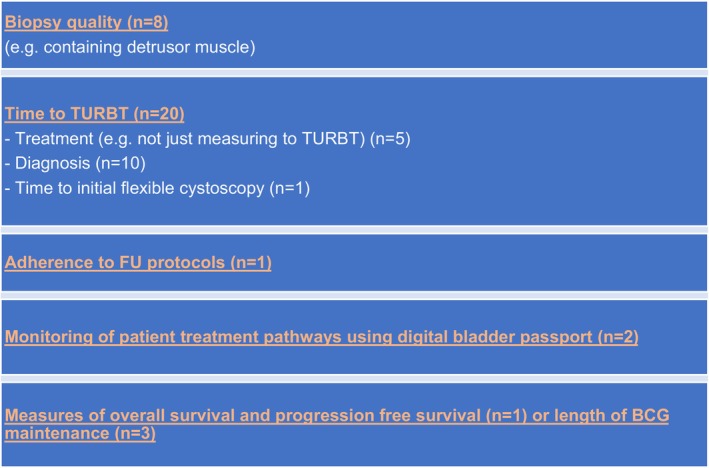
Common themes relating to important areas to audit or QPI to drive improvements in bladder cancer service reported by HCPs.

From the thematic analysis, insights into practice variability, resource challenges and unmet needs were identified. Common QPI/audit priorities reported included biopsy quality (*n* = 8), time to TURBT (*n* = 20), adherence to follow‐up protocols (*n* = 1), monitoring of patient treatment pathways using a digital bladder passport (*n* = 2) and measures of overall survival and progression‐free survival (*n* = 1) or length of BCG maintenance (*n* = 3) (Figure 4).

**FIGURE 5:**
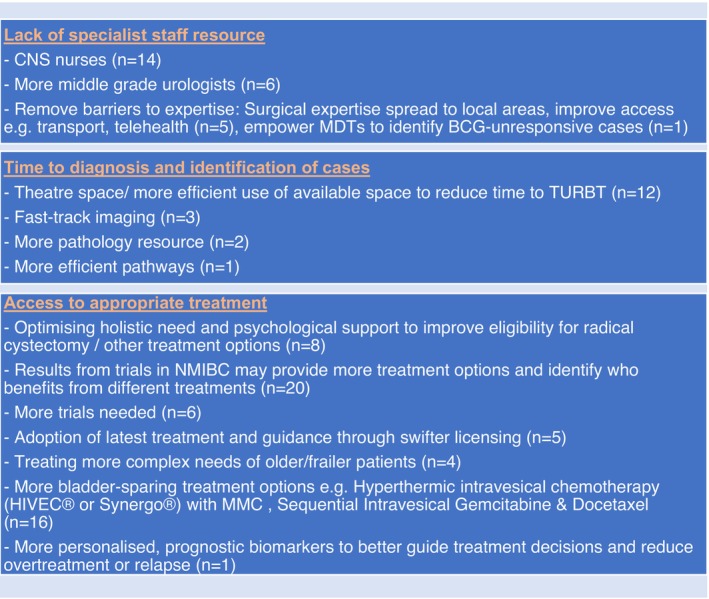
Common themes relating to important unmet needs within the HR‐NMIBC pathway to be addressed in the next 12–18 months.

Common themes relating to important unmet needs within the HR‐NMIBC pathway to be addressed in the next 12–18 months are summarised in Figure 5.

Additional support needs for HCPs and patients are summarised in Table S3.

## 4 DISCUSSION

This national descriptive analysis provides the first ever clinician‐led, comprehensive mapping of the real‐world HR‐NMIBC care pathway across NHS regions. Variability in diagnostic timelines, BCG maintenance treatment duration and access to evidence‐based bladder‐sparing treatment and clinical trial options were demonstrated. The findings from this expert‐led cross‐sectional study highlight persistent unmet clinical needs and therefore associated opportunities for optimisation of future HR‐NMIBC NHS treatment and patient pathways.

The study demonstrates that, although most NHS centres adhere to NICE referral criteria and predominantly rely on EAU guidelines for HR‐NMIBC management, actual practice varied, and use of GIRFT recommendations was less frequently reported.^21^ HCPs reported that only 40.0% of patients typically receive a confirmed diagnosis within 6 weeks of referral, 1.4% within 4 weeks and 11.4% experience delays beyond 8 weeks, underscoring ongoing opportunities for diagnostic pathway optimisation. Expert respondents highlighted that optimisation of diagnostic pathways requires resource constraints, such as limited theatre space for TURBT and insufficient specialist nurse capacity to be addressed. This is consistent with previous reports of workforce shortages impacting cancer care delivery in the NHS.^6^, ^22^, ^23^

BCG induction and maintenance remain the mainstay adjuvant therapy,^20^ yet reported maintenance duration and completion rates were variable. HCPs reported that typically a median of 20.0% of patients complete induction plus at least 2 years of maintenance, whereas a median of 60.0% tend to complete BCG induction followed by up to 1 year of maintenance. These rates show that UK patients typically tend to receive more than the minimum level for ‘adequate BCG’ (as defined by the FDA)^11^ with up to 1 year of maintenance therapy completed more commonly than the recommended duration of up to 3 years.^6^, ^7^, ^8^ This may be reflective of the reported real‐world challenges such as patient toxicity, logistical barriers and historic supply shortages. The lack of systematic tracking of BCG outcomes and response categories (unresponsive, experienced and intolerant) further limits the ability to optimise care and benchmark against international standards.^6^, ^7^

In the BCG‐unresponsive HR‐NMIBC setting, HCPs reported that typically only 53.4% of patients are eligible and consent to RC, with 22.0% not proceeding with RC despite being eligible and the remaining 24.6% being ineligible. The reported patient‐centred concerns of RC, such as bladder preservation preference (98.6%), safety/morbidity (91.4%) and quality‐of‐life concerns (75.7%), dominate decision‐making and reflect findings from prior studies on RC consent and the unmet need for alternative bladder‐sparing treatment options.^9^, ^10^ However, the NHS currently offers limited evidence‐based bladder‐sparing options, and notably, further BCG therapy was considered to be most appropriate by 60.0% of HCPs for BCG‐unresponsive patients, despite limited efficacy in this setting.^24^ Access to hyperthermic intravesical chemotherapy (typically either HIVEC® or Synergo®) or clinical trials is inconsistent and often requires referral to tertiary centres.

A strength of this study is its national representation, capturing perspectives from 70 NHS healthcare professionals across 20 Cancer Alliances and all UK nations. The inclusion of key MDT respondents routinely engaged in HR‐NMIBC care, such as consultant urologists, specialist nurses, trainee urologists and clinical oncologists, enabled a nuanced and authoritative understanding of current MDT structures and resource challenges in the UK. The mixed‐methods approach, combining quantitative and qualitative data, provides depth to the analysis of practice variability, areas for service development and identification of unmet needs.

However, several limitations must be acknowledged. Expert respondents either consulted locally available databases to inform their responses or provided estimates based on their professional experience where databases were incomplete or unavailable. The reliance on self‐reported data therefore introduces potential recall bias and may not fully capture local database granularity. The cross‐sectional design offers a single time‐point snapshot, limiting assessment of temporal trends or the impact of more recent policy changes. Selection bias may be present due to voluntary participation, and regional differences in resources and practice may affect generalisability. Additionally, the absence of patient‐level outcome data restricts the ability to directly correlate pathway inefficiencies with clinical endpoints.

### 4.1 Implications for clinical practice and future research

These findings underscore an urgent need to standardise HR‐NMIBC care pathways across the NHS. Seventy per cent of healthcare professionals reported insufficient specialist nurse capacity to meet anticipated demands for HR‐NMIBC care. Investment in dedicated bladder‐cancer specialist nursing teams (underpinned by clear role definitions, protected funding and succession planning) may therefore be warranted to support core pathway functions, including holistic needs assessment, administration of intravesical therapy, timely treatment review and ongoing cystoscopic and cytological surveillance, thereby capacitating consistent, guideline‐concordant care. Enhanced nurse education and support, reported by 55.3% of HCPs, could further strengthen care delivery.

The reported delays in time to diagnosis and to TURBT indicate opportunities for potential pathway optimisation across the UK. Respondents reported three principal bottlenecks including triage protocol development, theatre capacity and pathology turnaround. As such, targeted interventions could improve time to definitive treatment and enable prioritisation of high‐risk cases. Practical measures include implementation of fast‐track referral pathways with standardised triage criteria, protected or dedicated theatre lists for suspected high‐risk cases and electronic patient‐tracking systems to monitor progress through the pathway and trigger escalation when delays occur. Regarding BCG maintenance and outcome tracking, standardisation of BCG protocols and systematic documentation of maintenance duration and response categories are critical for benchmarking and service improvement. Integration of QPIs and a digital on‐treatment patient self‐assessment record (‘bladder passports’) may facilitate more accurate tracking and audit.

These findings emphasise a critical gap in evidence‐based bladder‐sparing treatments for patients with BCG‐unresponsive disease. Viable alternatives to RC, particularly for frail and older patients, are needed, and there is an urgent need to expand clinical trial networks, strengthen trial infrastructure and enable broader access to evidence‐based bladder‐sparing treatment options. Further research in these areas could accelerate generation of high‐quality evidence and improve treatment choices for future patients.

Universal agreement among all respondents on the value of a national bladder cancer audit reflects the need for standardised quality metrics and evidence‐based recommendations. Such initiatives would reduce regional variation, support service improvement and inform future research priorities.

Despite mapping current practice, several unanswered questions remain. The impact of diagnostic and treatment delays on long‐term oncological outcomes warrants further investigation, ideally through linkage with patient‐level registry data. The effectiveness and safety of emerging bladder‐sparing therapies in the UK population, particularly for patients ineligible for RC, should be evaluated in prospective studies. Additionally, strategies to address workforce shortages, such as advanced nurse practitioner roles, telehealth and regional MDT collaboration, require further exploration.

## 5 CONCLUSION

In summary, this cross‐sectional national assessment highlights the urgent requirement for pathway optimisation in HR‐NMIBC care within the NHS. Key priorities include enhancing specialist nurse resourcing, streamlining diagnostic and treatment pathways, standardising BCG protocols and outcome tracking and expanding access to evidence‐based bladder‐sparing treatments and clinical trials. These measures are critical to improving patient outcomes, reducing national disparities and meeting the ongoing unmet clinical needs of HR‐NMIBC patients across the UK.

## AUTHOR CONTRIBUTIONS

Jonathan Aning, Bernadett Szabados and Stephen McCormack had full access to all the data in the study and take full responsibility for the integrity of the data and the accuracy of the data analysis. *Study concept and design*: Jonathan Aning, James W. F. Catto, Rebecca Martin, Kathryn Chatterton, Paramananthan Mariappan, Bernadett Szabados. *Acquisition of data*: Stephen McCormack. *Analysis and interpretation of data*: Jonathan Aning, James W. F. Catto, Rebecca Martin, Kathryn Chatterton, Paramananthan Mariappan, Bernadett Szabados. *Drafting of the manuscript*: Edward Ottley, Joseph Hickey, Stephen McCormack. *Critical revision of the manuscript for important intellectual content*: Jonathan Aning, James W. F. Catto, Rebecca Martin, Kathryn Chatterton, Paramananthan Mariappan, Edward Ottley, Joseph Hickey, Stephen McCormack, Simran Gill, Bernadett Szabados. *Obtaining funding*: Stephen McCormack, Simran Gill. *Supervision*: Jonathan Aning. *Other*: None.

## CONFLICT OF INTEREST STATEMENT

Jonathan Aning has received honoraria for advisory boards or providing medical education from Accord, AstraZeneca, Astellas, Bayer, Curium, Janssen, Merck and Nonacus and has received institutional research funding from Nonacus. James W.F. Catto has received consulting fees from AstraZeneca, Ferring, Ipsen, Roche and Janssen; has received speaker fees from Bristol Myers Squibb, Pfizer, Merck Sharp & Dohme, Janssen, Astellas, Nucleix, InMed and Roche; has received honoraria for membership in advisory boards from Ferring, Roche, Gilead, Photocure, Pfizer, Bristol Myers Squibb, QED Therapeutics and Janssen; and has received institutional research funding from Roche. Rebecca Martin has received consultancy fees ± honoraria from Medac pharma, J&J and Pfizer. Kathryn Chatterton has received speaker fees from Medac pharma, J&J and Photocure and has received honoraria for advisory boards from Medac pharma and Photocure. Paramananthan Mariappan has received honoraria and consulting fees from Baxter, BMS, Coloplast, Ethicon, Janssen, Medac Pharma, Nucleix, Pfizer, Photocure and Storz. Edward Ottley is an employee of OPEN Health Communications LLP. Joseph Hickey is an employee of OPEN Health Communications LLP. Stephen McCormack is an employee of Johnson & Johnson Innovative Medicine. Simran Gill is an employee of Johnson & Johnson Innovative Medicine. Bernadett Szabados has received speaker fees from Merck, Janssen, Photocure, Astellas, Ferring and consulting/advisory fees from Pfizer, Astellas, Photocure and Johnson & Johnson.

## Supporting information


**Table S1:** Roles and responsibilities of respondents.
**Table S2:** Other roles reported by HCPs.
**Table S3:** Additional support needs for HCPs and patients.

## Data Availability

The datasets generated during and/or analysed during the current study are not publicly available but are available from the corresponding author on reasonable request.
