# Supplementary material for: State of the nation: Understanding the current NHS treatment pathway to identify opportunities to advance future care of patients with high‐risk non‐muscle invasive bladder cancer in the UK (SPAN‐UK)
Source: BJUI Compass. 2026 Jun 28;7(7):e70236. doi: 10.1002/bco2.70236 (PMC13310956; doi:10.1002/bco2.70236)
Supplement: Supplementary file 1 — Table S1: Roles and responsibilities of respondents. Table S2: Other roles reported by HCPs. Table S3: Additional support needs for HCPs and patients. [file BCO2-7-e70236-s001.docx]

**S**tate of the nation**:** Understanding the current NHS treatment **P**athway to identify opportunities to **A**dvance future care of patients with high-risk **N**on-muscle invasive bladder cancer in the **UK** (**SPAN-UK**)

**Authors:** Jonathan Aning^1^, James WF Catto^2^, Rebecca Martin^3^, Kathryn Chatterton^4^, Paramananthan Mariappan^5^, Edward Ottley^6^, Joseph Hickey^6^, Stephen McCormack^7^, Simran Gill^7^, Bernadett Szabados^8^.

**SUPPLEMENTARY INFORMATION**

**Supplementary tables**

**Table S1: Roles and responsibilities of respondents**

| **Who, within respondent's MDT, takes a lead role at each of the following parts of the NMIBC pathway?** | **Consultant Urologist** | **Staff Grade/ Middle Grade Urologist** | **Trainee Urologist** | **Uro-oncology Surgical Fellow** | **Medical Oncologist** | **Clinical Oncologist** | **Staff Grade/ Middle grade Oncologist** | **Nurse lead: CNS Nurse lead practitioner/ Dedicated bladder cancer CNS** | **Surgical Care Practitioner (SCP)** | **Histopathologist/ Pathologist** | **Pharmacy lead** | **Psychologist/ Psychiatrist** | **Other** ^#^ |
| --- | --- | --- | --- | --- | --- | --- | --- | --- | --- | --- | --- | --- | --- |
|  | N respondents, '% (n = 70) | N respondents, '% (n = 70) | N respondents, '% (n = 70) | N respondents, '% (n = 70) | N respondents, '% (n = 70) | N respondents, '% (n = 70) | N respondents, '% (n = 70) | N respondents, '% (n = 70) | N respondents, '% (n = 70) | N respondents, '% (n = 70) | N respondents, '% (n = 70) | N respondents, '% (n = 70) | N respondents, '% (n = 70) |
| Initial Diagnostic Flexible cystoscopy | 45 (64.3%) | 56 (80.0%) | 24 (34.3%) | 12 (17.1%) | 0 (0.0%) | 0 (0.0%) | 0 (0.0%) | 43 (61.4%) | 5 (7.1%) | 0 (0.0%) | 0 (0.0%) | 0 (0.0%) | 9 (12.9%) |
| Biopsy and TULA (Transurethral Laser Ablation) for suspicious lesions. | 68 (97.1%) | 40 (57.1%) | 16 (22.9%) | 8 (11.4%) | 0 (0.0%) | 0 (0.0%) | 0 (0.0%) | 13 (18.6%) | 1 (1.4%) | 0 (0.0%) | 0 (0.0%) | 0 (0.0%) | 12 (17.1%) |
| Trans urethral removal of bladder tumour (TURBT) | 70 (100.0%) | 51 (72.9%) | 23 (32.9%) | 12 (17.1%) | 0 (0.0%) | 0 (0.0%) | 0 (0.0%) | 0 (0.0%) | 0 (0.0%) | 0 (0.0%) | 0 (0.0%) | 0 (0.0%) | 8 (11.4%) |
| Risk Stratification | 70 (100.0%) | 2 (2.9%) | 1 (1.4%) | 2 (2.9%) | 0 (0.0%) | 2 (2.9%) | 0 (0.0%) | 5 (7.1%) | 0 (0.0%) | 2 (2.9%) | 0 (0.0%) | 0 (0.0%) | 34 (48.6%) |
| Repeat TURBT (if indicated: e.g., high-grade, T1, or incomplete resection) or (if indicated based on risk and histology) | 70 (100.0%) | 48 (68.6%) | 23 (32.9%) | 11 (15.7%) | 0 (0.0%) | 0 (0.0%) | 0 (0.0%) | 0 (0.0%) | 0 (0.0%) | 0 (0.0%) | 0 (0.0%) | 0 (0.0%) | 8 (11.4%) |
| Holistic Needs Assessment | 5 (7.1%) | 2 (2.9%) | 2 (2.9%) | 2 (2.9%) | 0 (0.0%) | 0 (0.0%) | 0 (0.0%) | 62 (88.6%) | 0 (0.0%) | 0 (0.0%) | 0 (0.0%) | 0 (0.0%) | 26 (37.1%) |
| Counselling the patient on adjuvant treatment options | 60 (85.7%) | 25 (35.7%) | 3 (4.3%) | 3 (4.3%) | 1 (1.4%) | 1 (1.4%) | 0 (0.0%) | 54 (77.1%) | 0 (0.0%) | 0 (0.0%) | 0 (0.0%) | 0 (0.0%) | 5 (7.1%) |
| Psychological Support | 1 (1.4%) | 0 (0.0%) | 0 (0.0%) | 0 (0.0%) | 0 (0.0%) | 0 (0.0%) | 0 (0.0%) | 58 (82.9%) | 0 (0.0%) | 0 (0.0%) | 0 (0.0%) | 16 (22.9%) | 51 (72.9%) |
| Smoking Cessation advice | 6 (8.6%) | 1 (1.4%) | 1 (1.4%) | 1 (1.4%) | 0 (0.0%) | 0 (0.0%) | 0 (0.0%) | 38 (54.3%) | 0 (0.0%) | 0 (0.0%) | 0 (0.0%) | 0 (0.0%) | 61 (87.1%) |
| Decision Making on adjuvant intravesical Therapy for HR-NMIBC | 70 (100.0%) | 4 (5.7%) | 2 (2.9%) | 3 (4.3%) | 0 (0.0%) | 5 (7.1%) | 0 (0.0%) | 15 (21.4%) | 0 (0.0%) | 0 (0.0%) | 0 (0.0%) | 0 (0.0%) | 12 (17.1%) |
| Instillation of adjuvant Intravesical Therapy for HR-NMIBC | 4 (5.7%) | 1 (1.4%) | 0 (0.0%) | 0 (0.0%) | 0 (0.0%) | 0 (0.0%) | 0 (0.0%) | 67 (95.7%) | 0 (0.0%) | 0 (0.0%) | 0 (0.0%) | 0 (0.0%) | 6 (8.6%) |
| HR-NMIBC Patient surveillance/ follow ups (monitoring for recurrence/progression | 46 (65.7%) | 41 (58.6%) | 18 (25.7%) | 6 (8.6%) | 0 (0.0%) | 0 (0.0%) | 0 (0.0%) | 56 (80.0%) | 0 (0.0%) | 0 (0.0%) | 0 (0.0%) | 0 (0.0%) | 13 (18.6%) |
| TURBT to remove recurrent NMIBC | 70 (100.0%) | 49 (70.0%) | 22 (31.4%) | 12 (17.1%) | 0 (0.0%) | 0 (0.0%) | 0 (0.0%) | 0 (0.0%) | 0 (0.0%) | 0 (0.0%) | 0 (0.0%) | 0 (0.0%) | 7 (10.0%) |
| Bladder Sparing Treatment options at NMIBC recurrence | 60 (85.7%) | 1 (1.4%) | 0 (0.0%) | 0 (0.0%) | 0 (0.0%) | 19 (27.1%) | 0 (0.0%) | 6 (8.6%) | 0 (0.0%) | 0 (0.0%) | 0 (0.0%) | 0 (0.0%) | 39 (55.7%) |
| Identification of suitable HR-NMIBC candidates for Clinical trials (newly diagnosed or recurrent) | 62 (88.6%) | 0 (0.0%) | 0 (0.0%) | 0 (0.0%) | 2 (2.9%) | 19 (27.1%) | 0 (0.0%) | 1 (1.4%) | 0 (0.0%) | 0 (0.0%) | 0 (0.0%) | 0 (0.0%) | 29 (41.4%) |
| ^#^Other responses: See Supplementary Table S2. | | | | | | | | | | | | | |

**Table S2: Other roles reported by HCPs**

| **N respondents** | **Initial Diagnostic Flexible cystoscopy** |
| --- | --- |
| 1 | Only the consultant nurse does this in addition to middle grades (not CNS/ANP). |
| 1 | Consultant Nurse does this (not CNS/ANP) |
| 1 | Clinical Oncologist is aware that the diagnostic pathway is consultant Urologist led but is unsure regarding wider urology team involvement |
| 1 | Advanced Nurse Practitioners only (not CNS) |
| 1 | CNS do checking flexible cystoscopies only. Some referral sites do have nurse led 2WW services |
| 1 | General urology fellows can lead as part of contract for robotic surgery |
| 1 | ANP led 1 stop clinic |
| 1 | trainees and specialist nurses typically under consultant supervision |
| 1 | Trainees are under consultant supervision |
|  |  |
| **N respondents** | **Biopsy and TULA (Transurethral Laser Ablation) for suspicious lesions.** |
| 1 | Only the consultant nurse does this in addition to TULA consultant urologist (not CNS/ANP). |
| 1 | Consultant Nurse does this (not CNS/ANP) |
| 1 | Consultant or consultant supervised middle grade |
| 1 | Clinical Oncologist is aware that the diagnostic pathway is consultant Urologist-led but is unsure regarding wider urology team involvement |
| 1 | Advanced Nurse Practitioner only, not CNS |
| 1 | 1 ANP also does TULA |
| 1 | Urologists lead and CNS sometimes do follow-ups, where needed |
| 1 | Please note that there is a CNS who does perform TULA under Consultant Urologist supervision. |
| 1 | Biopsying is not done by TULA in this centre. |
| 1 | Biopsying is not done by TULA here. Samples are collected either during cystoscopy or TURBT. |
| 1 | Trainees typically under consultant supervision |
| 1 | Consultant supervised trainees |
|  |  |
| **N respondents** | **Trans urethral removal of bladder tumour (TURBT)** |
| 1 | Consultant or consultant supervised middle grade |
| 1 | Clinical Oncologist is aware that the diagnostic pathway is consultant Urologist led but is unsure regarding wider urology team involvement |
| 1 | Consultant Urologist supervised |
| 1 | Middle grades would be under consultant supervision |
| 1 | All consultant supervised |
| 1 | Non consultant urology team members are supervised by consultant when conducting TURBT |
| 1 | Trainees typically under consultant supervision |
| 1 | Consultant-supervised trainees |
|  |  |
| **N respondents** | **Risk Stratification** |
| 1 | MDT consensus led by Urologist and with CNS plus wider MDT members input |
| 1 | MDT consensus led by consultant |
| 21 | MDT consensus |
| 1 | MDT consensus. Note In teaching hospitals the surgical fellow may be fully qualified than in DGH setting. |
| 1 | Nurse led assessment within a sub MDT NMIBC discussion. CNS to discuss with urologist |
| 1 | MDT is Urologist led |
| 4 | Urologist-led MDT consensus |
| 1 | Urologist led MDT consensus informed by imaging, pathology report etc. |
| 1 | Urologist-led MDT decision where pathology input is key |
| 3 | Urologist led MDT consensus |
| 1 | Urologist led MDT consensus informed by input across specialties. |
| 1 | MDT consensus led by Urologist |
|  |  |
| **N respondents** | **Repeat TURBT (if indicated: e.g., high-grade, T1, or incomplete resection) or (if indicated based on risk and histology)** |
| 1 | Consultant or consultant supervised middle grade |
| 1 | Clinical Oncologist is aware that the that decision making here is Consultant Urologist led but is unsure regarding wider urology team involvement |
| 2 | Consultant supervised |
| 1 | Middle grades would be under consultant supervision |
| 1 | Non-consultants supervised by consultant |
| 1 | Trainees typically under consultant supervision |
| 1 | Trainees under consultant supervision |
|  |  |
| **N respondents** | **Holistic Needs Assessment** |
| 1 | Cancer nurse support workers do much of this by phone clinic |
| 1 | All urology nurses can do holistic needs. Also, there is a dedicated bladder CNS full time and another part time dedicated bladder CNS. |
| 1 | Also, Urology support workers can do this |
| 1 | Urology Support workers lead on most NMIBC holistic needs. CNS lead for HR-NMIBC |
| 1 | Most holistic needs assessments are conducted by the 2 Urology support workers. Unless for complex cases (where CNS would conduct the holistic needs assessment) |
| 1 | This is more typically done by CNS rather than Advanced Nurse Practitioners. |
| 1 | Cancer care coordinators do Holistic needs assessment and refer to CNS for care planning |
| 1 | Cancer Support Workers can do holistic needs assessments. |
| 1 | Cancer support workers (these are nurses employed by the Trust now, having previously been Macmillan) |
| 1 | Trust level access also to Support workers (nurses) |
| 2 | Macmillan support officers |
| 1 | Band 4 Support workers |
| 1 | Insufficient CNS so holistic needs assessments are not typically done. |
| 1 | Lack of specialist CNS resourcing means that this is currently lacking |
| 1 | Macmillan support officers |
| 1 | Macmillan support |
| 1 | Cancer coordinators increasingly support this too |
| 1 | MacMillan support workers/navigators support now available for conducting holistic needs assessments. |
| 1 | MacMillan Cancer Support Worker Led in consultation with CNS team |
| 1 | This is Macmillan holistic needs support worker led with CNS supervision/dialogue |
| 1 | Not always performed but would be done by CNS |
| 1 | Cancer support workers do this |
| 1 | Limited specialist nurse resourcing is a current challenge regionally and has been identified as something for resolution. |
| 1 | This is challenged by insufficient CNS headcount currently |
| 1 | Through Macmillan in association with CNS |
|  |  |
| **N respondents** | **Counselling the patient on adjuvant treatment options** |
| 1 | Depending on risk consultant if High Risk |
| 1 | Depending on risk consultant if High Risk |
| 1 | Cancer support workers refer to CNS for further psychological support needs planning |
| 1 | These discussions would usually be at Haematuria clinics and would equally be led by specialist nurses, middle grade urologists or consultant urologists. |
| 1 | The Haematuria clinics are equally led by these 3 job roles |
|  |  |
| **N respondents** | **Psychological Support** |
| 1 | CNS can signpost patients to further psychological support, if needed. |
| 1 | As part of holistic needs, nurses provide some support but there is no specific psychologist support additionally available, if needed currently. |
| 1 | Can refer for more psychological support, if needed. |
| 1 | No specific psychologist support. CNS would need to refer for support, if needed. |
| 1 | There is further psychological support (non-cancer specific) available which can be requested but access in the community can be variable. |
| 1 | urology support workers and CNS can assess psychological aspects during holistic needs assessment. There is additional support available, if needed (non-cancer specific) |
| 1 | Urology support workers and CNS can signpost to further psychological support, if needed |
| 1 | CNS can refer patients following holistic needs assessment for psychological support or smoking cessation services etc. |
| 1 | CNS can refer for further psychological support, if needed. |
| 1 | Maggie’s cancer can provide this if requested by CNS |
| 1 | Typically, CNS-led as part of holistic needs assessment. Patients who require further support can be referred to a psychologist where needed |
| 1 | CNS team are trained to level 1 and 2 as part of holistic needs with respect to psychological aspects. They can refer patients who require further support to psychologist (not bladder specific). |
| 1 | Cancer care coordinators could refer to Maggie’s/Macmillan if needed via CNS referral |
| 1 | CNS can signpost to psychological support, where needed. |
| 1 | By CNS as part of holistic needs. When required, the CNS can signpost patients to additional support. |
| 1 | further psych support (non-cancer specific) support groups can be requested, if needed |
| 1 | Non-cancer specific psych support is available if needed |
| 1 | Same as holistic needs so, CNS or cancer support worker-led and referral for further psychological support, if needed but funding challenges do exist at Trust level. |
| 1 | funding challenges to accessing trust level psych support. variable community access |
| 1 | Macmillan can signpost to support |
| 1 | Access to counselling available |
| 1 | Macmillan care navigators can sign post patients to support |
| 1 | General hospital access |
| 1 | Further psychological support can be requested, if needed but access is variable |
| 1 | Psych support variable in community |
| 1 | Macmillan care navigators can sign post to available psychological support |
| 1 | Psychology support is at trust level but is variable in the community |
| 1 | Insufficient CNS resource locally and access to community psych support is variable at best. |
| 1 | Lack of CNS resourcing means that this is currently lacking |
| 1 | Psych support available on request |
| 1 | Unaware of what psychological support services may be available at trust level. CNS-led holistic needs assessments could highlight cases in need of referral to any psychological support services that may exist either at Trust level or in the community. |
| 1 | Maggie’s cancer can provide this if requested |
| 1 | CNS team members conduct holistic needs assessments, from this, CNS team members can signpost patients that require psychological support to any available options. |
| 1 | Can be access to a Macmillan psych support service or community |
| 1 | Macmillan |
| 1 | In trust of localised psych support can be requested |
| 1 | CNS can refer patients who need psychological support to Macmillan services |
| 1 | CNS team members and MacMillan support workers/navigators can direct patients for further support if needed. |
| 1 | CNS can signpost patients for further psychological support via MacMillan cancer patient service |
| 1 | CNS can direct patients for further support where needed and if available. |
| 1 | CNS can refer patients for further support, if needed but the service is stretched/patchy |
| 1 | As part of holistic needs, CNS do a psychological evaluation. If further psychological support is needed there is access to psychology expertise. |
| 1 | CNS led referrals where needed for psychologist |
| 1 | Psychologist post currently vacant at trust level. |
| 1 | CNS team members can signpost patients to further support |
| 1 | Specialist nurses can refer patients for further psychological support if need is identified during holistic needs assessment. |
| 1 | Specialist Nurse key worker can signpost patients to psychologist or to a Patient Advisory Group or to Maggie’s Centres in NHS Scotland, these can provide free, comprehensive psychological and emotional support to anyone affected by cancer, if needed. |
| 1 | CNS can refer patient for further psychological support if this need is picked up during holistic needs or as key worker |
| 1 | Limited specialist nurse resourcing is a current challenge regionally and has been identified as something for resolution. |
| 1 | Caveat is that there is insufficient CNS headcount currently. However, it would be the CNS who would direct patients to further psychological support, where needed (as identified as part of the holistic needs assessment). |
| 1 | CNS/MacMillan can refer patients for further support, where available. |
|  |  |
| **N respondents** | **Smoking Cessation advice** |
| 1 | Smokers are advised and referred to local services by CNS |
| 1 | Nursing teams advise on smoking cessation and signpost patients to services available as part of the holistic needs. |
| 1 | CNS provide advice and refer patients to available services near where they live. |
| 1 | CNS can direct patients to smoking cessation services which are available. |
| 1 | variable community access can be signposted via primary care. |
| 1 | CNS team and urology support workers can direct patients to services in the community |
| 1 | Urology support workers and CNS team signpost patients to smoking cessation services. |
| 1 | The 2 Urology support workers would signpost patients to smoking cessation services during holistic needs. Unless complex cases where CNS would signpost to smoking cessation services during holistic needs assessment. |
| 1 | Smoking cessation services are available |
| 1 | Sign posted to available service (not bladder dedicated |
| 1 | Patients can be signposted to community smoking cessation services. |
| 1 | Patients get signposted by CNS to community support services that are available variably |
| 1 | Patients can access smoking cessation services in the community via their GP. |
| 1 | CNS can refer patients to community based smoking cessation services via GPs |
| 1 | CNS can refer patients to community support services via GPs |
| 1 | Variable community access |
| 1 | Smoking cessation services are available but are dependent on patients wanting this |
| 1 | No smoking cessation service available at trust level. Variable community access and subject to GP signposting. |
| 1 | no longer available at trust level. So, GP signposting to community access is currently all that can be done. |
| 1 | Community services can be signposted to patients |
| 1 | No hospital-based service. GP may be able to signpost patients to community services for smoking cessation. |
| 1 | Respondent unaware of Smoking cessation service availability. Possible community access? |
| 1 | Smoking cessation services available in the community |
| 1 | Access to Community based smoking cessation |
| 1 | Community access to smoking cessation services |
| 1 | Can be requested. Some community smoking cessation services are available. |
| 1 | There is smoking cessation services available for CNS to refer patients to |
| 1 | available in community |
| 1 | Access via community |
| 1 | Smoking cessation variable following GP referral |
| 1 | Smoking cessation advice services availability community only and variable. |
| 1 | community based smoking cessation services are available variably |
| 1 | Smoking cessation service available in the Trust |
| 1 | Smoking cessation service available at the Hospital |
| 1 | smoking cessation services available on request |
| 1 | smoking cessation services available in community setting or via Macmillan |
| 1 | There are smoking cessation services available locally to where patients live and within Trust |
| 1 | General hospital access |
| 1 | Sign posted to available service (not bladder dedicated) |
| 1 | Smoking cessation service available in Trust and can be locally available |
| 1 | Access to Community based y smoking cessation |
| 1 | Community Access to Community based y smoking cessation |
| 1 | All selected team members share leadership on smoking cessation advice but there are community based services and trust level support available via Macmillan etc. |
| 1 | Not readily available at Trust but some patchy community coverage |
| 1 | CNS team members and MacMillan support workers/navigators can direct patients for further support if needed. |
| 1 | CNS can signpost patients for smoking cessation services available in the community |
| 1 | CNS can refer patients to services via GP |
| 1 | CNS led referral, where needed to any available service in the community |
| 1 | CNS team can signpost patients to services available locally |
| 1 | CNS direct patients to available services during holistic needs assessments |
| 1 | CNS can refer to smoking cessation service |
| 1 | there are some services available for patients and CNS can signpost. |
| 1 | There is no smoking cessation service at the hospital, but CNS team can signpost patients to community services, where available, |
| 1 | Note that smoking cessation services are community based only |
| 1 | Again, Specialist nurses can signpost smokers to cessation services available in the community |
| 1 | Key worker can support patients’ access to community smoking cessation services where available. |
| 1 | Specialist nurse can refer to smoking cessation services in the community |
| 1 | Community based Smoking cessation services can be signposted to patients by key worker |
| 1 | Limited specialist nurse resourcing is a current challenge regionally and has been identified as something for resolution. |
| 1 | Caveat is that there is insufficient CNS headcount currently. However, it would be the CNS who would direct patients to further support (community based smoking cessation), where needed (as identified as part of the holistic needs assessment). |
| 1 | CNS/MacMillan can refer patients for further support, where available but access to smoking cessation has become more variable/challenging due to budget cuts. |
|  |  |
| **N respondents** | **Decision Making on adjuvant intravesical Therapy for HR-NMIBC** |
| 1 | Bladder pathway meeting consultant led in consultation with nurses and radiology |
| 5 | MDT consensus |
| 1 | MDT consensus. CNS consulted |
| 1 | CNS/ANP can help Consultant Urologist decision making with patient level context/insights since they are the main point of patient contact. |
| 1 | Intravesical consenting is urologist led |
| 1 | MDT discussion |
| 1 | Discussed at MDT |
| 1 | Urologist led MDT consensus |
|  |  |
| **N respondents** | **Instillation of adjuvant Intravesical Therapy for HR-NMIBC** |
| 1 | Band 5 CNS team members |
| 1 | This is Advanced Nurse Practitioner only (not CNS) |
| 1 | Consultants would instil chemo e.g. MMC and CNS would instil BCG |
| 1 | Non-bladder dedicated Uro-Oncology outpatient nurses do the instillations of adjuvant intravesical therapy for HR-NMIBC in this trust |
| 1 | This is actually done by non-bladder specialist Uro oncology outpatient nurses. One ANP does do HIVEC, but this would be for BCG unresponsive cases. |
| 1 | Limited specialist nurse resourcing is a current challenge regionally and has been identified as something for resolution. |
|  |  |
| **N respondents** | **HR-NMIBC Patient surveillance/ follow ups (monitoring for recurrence/progression** |
| 1 | Physician Associates (PAs) also play a role here working as part of the multidisciplinary team to provide support with surveillance aspects of patient management |
| 1 | Consultant Nurse led |
| 1 | consultant nurse led surveillance |
| 1 | CNS trained to perform cystoscopies and middle grades |
| 1 | whoever does the flexible cystoscopies |
| 1 | This is currently a Capacity issue |
| 1 | ANP only (not CNS) |
| 1 | Consultants lead on the higher risk cases and where anaesthetic is needed. CNS team do the rest. |
| 1 | whoever is doing the flexible cystoscopies would lead on the monitoring for recurrence |
| 1 | whoever does the checking flexible cystoscopies. This would more often be the CNS. |
| 1 | Combination approach. Urologist led phone follow up every 6 months. |
| 1 | Specialist nurses and trainees would be under consultant supervision. |
| 1 | Trainees would be under consultant supervision |
|  |  |
| **N respondents** | **TURBT to remove recurrent NMIBC** |
| 1 | Consultant or consultant supervised middle grade |
| 1 | Clinical Oncologist is unaware to what extent the middle grade urologists get involved here. |
| 1 | Middle grades would be under consultant supervision |
| 1 | all under consultant supervision |
| 1 | Non-Consultant urologists would be under consultant supervision. |
| 1 | Trainees under consultant supervision |
| 1 | Trainees would be under supervision |
|  |  |
| **N respondents** | **Bladder Sparing Treatment options at NMIBC recurrence** |
| 1 | Consultant refers these cases for discussion at specialist MDT meeting |
| 1 | These Cases are Referred to specialist multidisciplinary meeting (SMDM) for discussion consensus on options/recommendations |
| 1 | Sent to tertiary referral site for consideration of options |
| 15 | MDT consensus |
| 1 | If not, further BCG we can refer these patients elsewhere |
| 1 | We refer these patients elsewhere for consideration of options including trials. |
| 1 | The local MDT would refer to Specialist MDT for consideration for bladder sparing treatment or a trial in this setting. |
| 1 | CNS/ANP are key to this decision due to better patient level knowledge. Also, ANP leads on HIVEC. |
| 1 | MDT discussion for possible referral |
| 1 | MDT discussion/consensus |
| 1 | MDT discussion and research team consulted on any open trials |
| 2 | Urologist led MDT consensus |
| 1 | All potential RC candidates are referred to the surgical site where this will be discussed with patients and thus some patients refuse and are referred back to CNS led service for BCG or MMC or consideration for trial |
| 1 | MDT discussion for possible referral |
| 1 | HIVEC (more straightforward) and SYNERGO (CIS?) |
| 1 | MDT consensus |
| 1 | Joint bladder clinic with oncologist and urologist and CNS(this is rare) |
| 1 | Refer for discussion at Specialist MDT |
| 1 | Referral for discussion at specialist MDT |
| 1 | These would be discussed at specialist MDT |
| 1 | This is 95% Urologist led but ~5% of the time clinical oncologists may seek involvement if there's a trial or radiotherapy is being utilised etc. |
| 1 | Specialist MDT decision (SMDT) run jointly with tertiary centre |
| 1 | These cases are discussed at the regional MDT during the bladder section |
| 1 | These cases are discussed at the regional MDT and consultant led consensus on treatment. |
|  |  |
| **N respondents** | **Identification of suitable HR-NMIBC candidates for Clinical trials (newly diagnosed or recurrent)** |
| 1 | There is only 1 urologist with a specialist interest in bladder cancer currently. The CNS team share all uro-oncology workload including bladder. So, this makes patient identification for specific bladder trials very challenging at times. |
| 1 | Depends on what trial it is. |
| 1 | MDT consensus |
| 1 | Depends on who the PI is |
| 1 | It depends on whether the trial is Oncologist or urologist led and whether research team members attend MDTs |
| 1 | trials team members |
| 1 | We don't run any trials locally but can refer. |
| 1 | Trials are done in a referral site so trial team members from there are key points of contact to identifying cases. |
| 1 | Ideally, a research team liaison should routinely attend MDT meetings to highlight trials that are open. It can be challenging to keep track of what trials are open, what the eligibility criteria are etc. |
| 1 | research team members and PI |
| 1 | Depends on PI. Also, there are research team members to assist with this. |
| 1 | Trials team and PI |
| 1 | Can refer to nearby teaching hospitals for trials |
| 1 | Depends on trial whether its Urologist or Clin Onc. There is also Research team members who support identification |
| 1 | PI and trials team |
| 1 | Trial PI and research team |
| 1 | Research trial group and nurses, |
| 1 | PI or research team |
| 1 | Research coordinator |
| 1 | research nurse |
| 1 | Depends on PI for open trials |
| 1 | Research trial group and nurses, |
| 2 | Depends on trial whether its Urologist or Clin Onc. There is also Research team members who support identification. |
| 1 | None active at the moment |
| 1 | depending on trial |
| 1 | Again, specialist MDT discussions on trial candidates. |
| 1 | Specialist MDT decision (SMDT) run jointly with tertiary centre |
| 1 | Depends on trial PI |
|  |  |
| **N respondents** | **Who, within your MDT, takes a lead role at each of the following parts of the NMIBC pathway?** |
| 1 | ANP plus Urology Registrar led |
| 1 | Consultant Nurse led Haematuria clinic |
| 1 | Consultant diagnostic nurse led |
| 1 | Trainee urologists/specialist registrars mostly lead these |
| 1 | Middle grade urologist led |
| 2 | Middle grade urologist team |
| 1 | Middle grade led but under urology consultant supervision |
| 1 | Uro-oncology CNS led with junior doctor support |
| 1 | Some are urologist led but most are urology middle grade led. |
| 1 | Some 2WW clinics are consultant-led. Others are Middle grade urologist-led. CNS involvement at diagnosis |
| 1 | Currently Registrar and ANP led service |
| 1 | Consultant urologist oversight plus 1 ANP and urology middle grades too |
| 2 | Uro-Oncology CNS and Urology Middle grade |
| 1 | Uro-Oncology CNS led |
| 1 | Nurse Consultant within Urology |
| 1 | Advanced Nurse Practitioner led service |
| 1 | 2 Advanced Nurse Practitioners co-lead with urology middle grades/registrars |
| 1 | Middle grade urologist and uro-oncology CNS-led |
| 1 | Staff Grade/middle grade urologist |
| 1 | Trainee/middle grade urologist led |
| 1 | Uro-oncology CNS (not bladder cancer dedicated) |
| 1 | Uro-oncology CNS led (no bladder dedicated CNS) |
| 1 | Middle grade urologist led under consultant urologist supervision |
| 1 | To account for the volume of cases and radiology capacity constraints, such clinics are run across the week for this broad referral area, and the leadership of the clinics is shared among the CNS team (4) and the lead urologists plus surgical fellow (3) |
| 1 | Three Band 7 bladder dedicated CNS team members lead the Haematuria clinics |
| 1 | Services are led by Urology Consultant, Urology Middle grade and Uro-Oncology CNS |
| 1 | Led by Specialist Urology Diagnostic Nurse |
| 1 | ~70% of Haematuria clinics are Urology middle grade led. 20% are Consultant urologist led and 10% are CNS led |
| 1 | Urology band 7 CNS |
| 1 | Booked into “general Urology” one stop clinic |
| 1 | ~33% each: ANP, Middle grade urologist and urology consultant led. More ANP led services may be possible in future subject to increased resourcing |
| 1 | In this region there is an equal split where clinics are led by Advanced Nurse Practitioner, Consultant Urologist, Middle grade Urologist |
|  |  |
| **N respondents** | **For patients with non-muscle invasive bladder cancer (NMIBC), how do you currently assess patient frailty?** |
| 1 | Geriatrician support for all 65+ year old cases |
| 1 | Geriatrician support for all 65+ year old cases. Proactive care of older patients in set up |
| 1 | WHO ECOG Performance status sometimes formally done |
| 9 | WHO ECOG Performance status |
| 1 | ECOG performance status too |
| 1 | WHO ECOG Performance Status is sometimes performed |
| 1 | WHO ECOG performance status is sometimes done. |
| 1 | WHO ECOG performance status is used plus Charleson comorbidity index is used for Radical Cystectomy cases only (to help with eligibility assessment). |
| 1 | Patients undergoing radical cystectomy may receive an additional cardiopulmonary exercise test (CPET) as part of their preoperative assessment. This test helps evaluate the patient's overall fitness and identify potential risks associated with surgery, particularly in older patients or those with comorbidities. |
| 1 | WHO ECOG performance status (formal/informal frailty assessments often already done at referring sites) |
| 1 | EORTC (European Organisation for Research and Treatment of Cancer) performance status is evaluated as part of the risk stratification of recurrent NMIBC patients |
| 1 | we would formally assess surgical candidates for frailty by using the American Society of Anaesthesiologists (ASA) physical status classification system. So, this is what we use ahead of any surgical procedure that requires anaesthesia. For example, Radical Cystectomy but also TURBT. |
| 1 | The American Society of Anaesthesiologists (ASA) physical status classification system is used locally as a grading system to determine patient frailty ahead of surgical procedures that require anaesthesia. So, this is done ahead of TURBT and Radical Cystectomy. |
| 1 | Charlson Comorbidity Index and CFS are used to assess eligibility for RC. Otherwise, frailty is assessed by clinical opinion. |
| 1 | Formal frailty criteria now routinely also performed by trust level geriatricians |
| 1 | In respondent's trust, the NHS has implemented a geriatrician-led frailty assessment service |
| 1 | WHO performance status is sometimes provided plus assessment at MDT of overall patient comorbidities etc. |
| 1 | WHO performance Status |
| 1 | We also use the WHO performance status to assess frailty |
| 1 | The EPIC system is available locally which is where a formal frailty assessment can be followed and recorded, if warranted. |
| 1 | At referral site for Radical Cystectomy, the pre assessment team do use the Rockwood frailty Scale, WHO ECOG PS is also sometimes used when assessing trial candidates and in other scenarios. |
| 1 | At referral site where Radical Cystectomies are performed, the pre assessment team do use a frailty index (respondent unsure which one), WHO ECOG Performance Status is also sometimes done |
| 2 | WHO ECOG PS and the American Society of Anaesthesiology (ASA) frailty score |
| 1 | sometimes WHO ECOG performance status gets recorded. |
| 1 | Comprehensive Geriatric Assessment (CGA)- This is done by POPS team |
| 1 | Clinical opinion/frailty assessment is currently variable depending on urologist assessor |
|  |  |
| **N respondents** | **At your centre, for BCG unresponsive NMIBC patients, which of the following bladder sparing treatment options tend to be considered most appropriate** |
| 1 | The sequential intravesical Gemcitabine & Docetaxel (Gem/Doce) isn't available yet but is being set up. Trials would be the next option but only for the fitter patients. |
| 1 | Or Epirubicin is sometimes used with hyperthermia (heat treatment). |
| 1 | The Di Stasi method, where Electromotive Drug Administration (EMDA) with MMC as an adjunct to further BCG is considered, in cases of BCG failure. Or hyperthermic intravesical chemotherapy (HIVEC) with epirubicin (as an option other than HIVEC with MMC) may offer another bladder-sparing alternative |
| 1 | Ongoing surveillance with cystoscopy or TULA |
| 1 | There are bladder sparing trials under consideration locally |
| 1 | Radiotherapy with a radiosensitiser |
| 1 | If possible, fitter patients might be referred elsewhere for any trials that may be open |
| 1 | Could be referred, if eligible, to another site for any bladder sparing trial that might be open (this has very rarely been achieved in reality, so far over the past year) |
| 1 | Referral of fitter candidates for consideration for Bladder Sparing Trials if open elsewhere |
| 5 | Bladder Sparing trials |
| 1 | In the process of setting up HIVEC access as part of a trial. |
| 1 | Further BCG is offered only if there is a long disease free interval after prior BCG. More commonly HIVEC is considered. Clinical trials are the preferred option for all eligible patients if SOC options like HIVEC are included as comparators. |
| 1 | Radiotherapy is sometimes offered in the bladder sparing setting. We are about to start using Sequential Intravesical Gem/Doce. Another option is Bladder sparing clinical trials |
| 1 | bladder sparing trials, if possible. |
| 1 | bladder sparing trials, if open. There's a lack of options here. |
| 1 | TULA is also offered locally (other than BCG reinduction). For HIVEC/trails patients would need to be referred elsewhere |
| 1 | Bladder Sparing Trials (HIVEC and trials are only available if patients are referred elsewhere). |
| 1 | Bladder Sparing Trials and HIVEC would both require referral elsewhere so this can lead to patients opting for further BCG if they are unwilling to go elsewhere for HIVEC or are not open to trials |
| 1 | bladder sparing trials are open in sites where patients can be referred. |
| 1 | intravesical epirubicin, is a potential bladder-sparing treatment option for those patients who are not candidates or prefer to avoid surgery. Epirubicin, either alone or in combination with other therapies, can offer a chance to preserve the bladder. |
| 1 | intravesical epirubicin after TURBT in BCG unresponsive NMIBC patients |
| 1 | Use SYNERGO to deliver hyperthermic MMC which is different tech to the HIVEC system |
| 1 | trial in referral area intravesical IO |
| 1 | Bladder sparing trial versus HIVEC, if open |
| 1 | Trial, if open, ideally versus HIVEC |
| 1 | other than further BCG or trials (if open) we tend to manage with endoscopic resections followed by intravesical therapy |
| 1 | generally, be managed with endoscopic resections followed by some form of intravesical therapy, for example epirubicin |
| 1 | Bladder sparing trial, if open and patient eligible. |
| 1 | Trial with novel intravesical device |
| 1 | trials where possible |
| 1 | trial if available |
| 1 | Access to RT as part of local studies/clinical trials |
| 1 | Bladder Sparing trial, if available |
| 1 | Hyperthermic Intravesical MMC using SYNERGO (not HIVEC) is the other option and is available at another site. |
| 2 | trials |
| 1 | trials, where possible |
| 1 | Trials where possible are also considered within an acceptable geographic range for patients. |
| 1 | Majority of such cases are considered for trials when possible. If not, then other options are considered. |
| 1 | This site utilises SYNERGO Hyperthermic MMC (not HIVEC) |
| 1 | Also have access to Hyperthermic MMC (SYNERGO) |
| 1 | Access to Hyperthermic MMC using SYNERGO tech (not HIVEC) at a nearby teaching hospital. |
| 1 | Access to Hyperthermic MMC is available in this trust but the SYNERGO tech is used not HIVEC |
| 1 | Radiotherapy is sometimes considered here. Also, there is some access locally to Hyperthermic MMC using the SYNERGO tech |
| 1 | Lack of options here is a real problem. Difficult to open trial and patients in this setting may struggle to meet eligibility criteria for trials. |
| 1 | Please note that sequential Gem/Doce is not yet available but is under discussion. Beyond this, clinical trials are the only remaining option that could be considered for the fitter patient etc. |
| 1 | We can refer some of the fitter patients for trials elsewhere if there are any open |
| 1 | Refer elsewhere for trials if open |
| 1 | referral elsewhere for bladder sparing trials, if open or TULA management or HIVEC. Note whilst HIVEC is 2nd most offered option after BCG re-induction, it does also require referral elsewhere. |
| 1 | Nurse led TULA service is available to manage disease burden for some patients who are ineligible for further BCG or HIVEC. Bladder sparing trials, if possible, for fitter patients. |
| 1 | Bladder Sparing trials are open at the moment |
| 1 | we can refer to tertiary centre if a trial is open (none at present). Note HIVEC is only available at tertiary referral site, and some patients are either frail or prefer to continue with treatment locally. |
| 1 | trials if open and patient eligible |
| 1 | refer to trials, where possible for fitter patients who are ok with travel and likely to meet eligibility criteria |
| 1 | Trials, whenever possible, are offered as options also |
| 1 | Sequential Intravesical Gem/Doce is currently in set up but is not yet available. There is no HIVEC or trial availability in set up for this setting for this region currently. |
| 1 | Sequential Intravesical Gem/Doce access is in set up but is not yet available |
| 1 | Surgical debulking with maintenance |

**Table S3: Additional support needs for HCPs and patients**

| **What additional support would be helpful for respondents as healthcare professionals (HCPs) in regard to managing and/or following up patients on new therapies** | **Number of respondents [N=47]** | **% (n =47)** |
| --- | --- | --- |
| Cancer Tracker | 0 | 0.0 |
| Allied Healthcare Professional educational support | 0 | 0.0 |
| Specialist Nurse educational support | 26 | 55.3 |
| Expert specialist HCP led Dedicated Bladder Cancer educational support | 26 | 55.3 |
| HCP adverse event management app | 1 | 2.1 |
| HCP internet portal | 0 | 0.0 |
| Home care support | 2 | 4.3 |
| Electronic/Digital HCP educational support | 0 | 0.0 |
| Paper HCP educational support | 1 | 2.1 |
| None needed | 3 | 6.4 |
| Other ^#^ | 9 | 19.1 |
| **In the respondent's opinion, what additional support would be helpful for patients in regard to managing and/or following up patients on new therapies?** | **N respondents** | **% (n =36)** |
| Electronic/Digital patient education | 10 | 27.8 |
| Patient app | 4 | 11.1 |
| Patient internet portal | 1 | 2.8 |
| Home care support | 7 | 19.4 |
| Paper patient education | 30 | 83.3 |
| QR codes on medication packaging | 5 | 13.9 |
| None needed | 1 | 2.8 |
| Other* | 1 | 2.8 |
| ^#^Other responses: ‘Support to attend congresses’ (n=1); ‘Congress attendance support for specialist nurse team members across the NHS’ (n=1); ‘Support for congress attendance’ (n=1); ‘Congress support’ (n=1); ‘More research collaboration opportunities’ (n=1); ‘Better databases across the NHS to provide data on HR-NMIBC and better evaluate service and outcomes’ (n=1); ‘Sponsorship to congresses’ (n=1); ‘Support of UK HCPs to attend some international congresses’ (n=1); ‘Support to attend international congresses such as EAU. More UK involvement in trials. More support from aspiring PIs in the UK’ (n=1).  *Other responses: ‘More research including UK patient voice’ (n=1). | | |

**Survey**

**SPAN-UK**

**S**tate of the Nation: Understanding the current NHS treatment **P**athway to identify opportunities to **A**dvance future **N**MIBC patient care in the **UK**

CP-528357

Date of Preparation: June 2025

This research has been initiated and funded by Johnson & Johnson

**INTRODUCTION**

Thank you for taking part in this survey.

The main aims of this survey are to:

- Characterize the current patient management and treatment pathway from diagnosis to follow-up of high-risk non-muscle invasive bladder cancer (HR-NMIBC)
- Identify the areas for optimisation with current standard of care treatment and patient pathway
- Understand the evolving multidisciplinary team (MDT) structure, roles and responsibilities for HR-NMIBC decision-making in the NHS

Please note that the answers you give will be kept confidential and will be combined with responses received from other UK participants. Neither you nor your institution will be identified when discussing or publishing the aggregated results from this survey.

**Section 1: Interview details**

| **Participant ID:** |  |
| --- | --- |

| **Interview date:** | DD | MM | YYYY |
| --- | --- | --- | --- |
|  |  |  |  |

| **MSL Interviewer initials** | SMC |
| --- | --- |

**Section 2: Respondent profile**

**Has the consent form been signed by the interviewee?**

☐Yes

☐No

**1. Please provide your Cancer Alliance** (select one option)

☐ Northern Cancer Alliance

☐ Lancashire and South Cumbria Cancer Alliance

☐ West Yorkshire and Harrogate Cancer Alliance

☐ Humber, Coast and Vale Cancer Alliance

☐ Cheshire and Merseyside Cancer Alliance

☐ Greater Manchester Cancer Alliance

☐ South Yorkshire and Bassetlaw Cancer Alliance

☐ West Midlands Cancer Alliance

☐ East Midlands Cancer alliance

☐ East of England – North Cancer Alliance

☐ East of England – South Cancer Alliance

☐ North Central London Cancer Alliance

☐ North East London Cancer Alliance

☐ RM Partners

☐ South East London Cancer Alliance

☐ Kent and Medway Cancer Alliance

☐ Surrey and Sussex Cancer Alliance

☐ Wessex Cancer Alliance

☐ Thames Valley Cancer Alliance

☐ Somerset, Wiltshire, Avon and Gloucestershire Cancer Alliance

☐ Peninsula Cancer Alliance

☐ Prefer not to say

☐ Other (please specify) ____________________________

**2. Please describe your main practice setting** (select one option)

☐ District general hospital

☐ University teaching hospital or Specialist Cancer Centre

☐ Other (please specify) ____________________________

**3A. Which of the following best describes your role in the care of patients with High-risk non-muscle invasive bladder cancer (HR-NMIBC)?**

☐ Specialist Nurse Team member (go to question 3B)

☐ Specialist Surgical team member (go to question 3C)

☐ Specialist Oncology team member (go to question 3D)

☐ Other bladder cancer team member (go to question 3E)

**3B. Please select which of the following describes your role as a Specialist Nurse Team member in the care of patients with HR-NMIBC**

☐ Advanced Nurse Practitioner (ANP)

☐ Dedicated Bladder Cancer Clinical Nurse Specialist (CNS)

☐ Urology Nurse Specialist

☐ Uro-Oncology Clinical Nurse Specialist (CNS)

☐ Enhanced Recovery CNS

☐ Clinical Nurse Specialist (CNS Multi-Specialty)

☐ Urology Trials Nurse

☐ Other (please specify)

**3C. Please select which of the following describes your role as a Specialist Surgical Team member in the care of patients with HR-NMIBC**

☐ Consultant Urologist

☐ Staff grade/middle grade urologist

☐ Uro-oncology Surgical Fellow

☐ Surgical care practitioner (SCP)

☐ Trainee urologist

☐ Other (please specify)

**3D. Please select which of the following describes your role as a Specialist Oncology Team member in the care of patients with HR-NMIBC**

☐ Medical Oncologist

☐ Clinical Oncologist

☐ Staff Grade/Middle grade Oncologist

☐ Other (please specify)

**3E. Please select which of the following describes your role as a bladder cancer team member in the care of patients with HR-NMIBC**

☐ Histopathologist/Pathologist

☐ Physician Associate

☐ Radiologist

☐ Oncology/Specialist pharmacist

☐ Other (please specify)

**4. Within the Hospital where you work, do you:**

**(please select which option best describes your local MDT where HR-NMIBC patients are discussed)**

☐ Have a Local uro-oncology MDT (with Bladder Cancer section)

☐ Have a dedicated Local MDT that only discusses Bladder Cancer patients

☐ Act as the Super Regional specialist Bladder Cancer MDT

☐ Other (please specify) ____________________________

**5. Who, within your MDT, takes a lead role at each of the following parts of the NMIBC pathway?** (Please select one for each pathway)

1. Initial Diagnostic Flexible cystoscopy

☐ Consultant Urologist

☐ Staff grade/ Middle grade Urologist

☐ Trainee Urologist

☐ Uro-oncology surgical fellow

☐ Med oncologist

☐ Trainee Urologist

☐ Clin oncologist

☐ Staff grade/ Middle grade oncologist

☐ Nurse lead: CNS Nurse lead practitioner/ Dedicated bladder cancer CNS

☐ Surgical care practitioner (SCP)

☐ Histopathologist/ Pathologist

☐ Pharmacy lead

☐ Psychologist/ Psychiatrist

☐ Other (please specify)

2. Biopsy and TULA (Transurethral Laser Ablation) for suspicious lesion

☐ Consultant Urologist

☐ Staff grade/ Middle grade Urologist

☐ Trainee Urologist

☐ Uro-oncology surgical fellow

☐ Med oncologist

☐ Trainee Urologist

☐ Clin oncologist

☐ Staff grade/ Middle grade oncologist

☐ Nurse lead: CNS Nurse lead practitioner/ Dedicated bladder cancer CNS

☐ Surgical care practitioner (SCP)

☐ Histopathologist/ Pathologist

☐ Pharmacy lead

☐ Psychologist/ Psychiatrist

☐ Other (please specify)

3. Transurethral resection of bladder tumour (TURBT)

☐ Consultant Urologist

☐ Staff grade/ Middle grade Urologist

☐ Trainee Urologist

☐ Uro-oncology surgical fellow

☐ Med oncologist

☐ Trainee Urologist

☐ Clin oncologist

☐ Staff grade/ Middle grade oncologist

☐ Nurse lead: CNS Nurse lead practitioner/ Dedicated bladder cancer CNS

☐ Surgical care practitioner (SCP)

☐ Histopathologist/ Pathologist

☐ Pharmacy lead

☐ Psychologist/ Psychiatrist

☐ Other (please specify)

4. Risk Stratification

☐ Consultant Urologist

☐ Staff grade/ Middle grade Urologist

☐ Trainee Urologist

☐ Uro-oncology surgical fellow

☐ Med oncologist

☐ Trainee Urologist

☐ Clin oncologist

☐ Staff grade/ Middle grade oncologist

☐ Nurse lead: CNS Nurse lead practitioner/ Dedicated bladder cancer CNS

☐ Surgical care practitioner (SCP)

☐ Histopathologist/ Pathologist

☐ Pharmacy lead

☐ Psychologist/ Psychiatrist

☐ Other (please specify)

5. Repeat TURBT (if indicated: e.g., high-grade, T1, or incomplete resection) or (if indicated based on risk and histology)

☐ Consultant Urologist

☐ Staff grade/ Middle grade Urologist

☐ Trainee Urologist

☐ Uro-oncology surgical fellow

☐ Med oncologist

☐ Trainee Urologist

☐ Clin oncologist

☐ Staff grade/ Middle grade oncologist

☐ Nurse lead: CNS Nurse lead practitioner/ Dedicated bladder cancer CNS

☐ Surgical care practitioner (SCP)

☐ Histopathologist/ Pathologist

☐ Pharmacy lead

☐ Psychologist/ Psychiatrist

☐ Other (please specify)

6. Holistic Needs Assessment

☐ Consultant Urologist

☐ Staff grade/ Middle grade Urologist

☐ Trainee Urologist

☐ Uro-oncology surgical fellow

☐ Med oncologist

☐ Trainee Urologist

☐ Clin oncologist

☐ Staff grade/ Middle grade oncologist

☐ Nurse lead: CNS Nurse lead practitioner/ Dedicated bladder cancer CNS

☐ Surgical care practitioner (SCP)

☐ Histopathologist/ Pathologist

☐ Pharmacy lead

☐ Psychologist/ Psychiatrist

☐ Other (please specify)

7. Counselling the patient on adjuvant treatment options

☐ Consultant Urologist

☐ Staff grade/ Middle grade Urologist

☐ Trainee Urologist

☐ Uro-oncology surgical fellow

☐ Med oncologist

☐ Trainee Urologist

☐ Clin oncologist

☐ Staff grade/ Middle grade oncologist

☐ Nurse lead: CNS Nurse lead practitioner/ Dedicated bladder cancer CNS

☐ Surgical care practitioner (SCP)

☐ Histopathologist/ Pathologist

☐ Pharmacy lead

☐ Psychologist/ Psychiatrist

☐ Other (please specify)

8. Psychological Support

☐ Consultant Urologist

☐ Staff grade/ Middle grade Urologist

☐ Trainee Urologist

☐ Uro-oncology surgical fellow

☐ Med oncologist

☐ Trainee Urologist

☐ Clin oncologist

☐ Staff grade/ Middle grade oncologist

☐ Nurse lead: CNS Nurse lead practitioner/ Dedicated bladder cancer CNS

☐ Surgical care practitioner (SCP)

☐ Histopathologist/ Pathologist

☐ Pharmacy lead

☐ Psychologist/ Psychiatrist

☐ Other (please specify)

9. Smoking Cessation advice

☐ Consultant Urologist

☐ Staff grade/ Middle grade Urologist

☐ Trainee Urologist

☐ Uro-oncology surgical fellow

☐ Med oncologist

☐ Trainee Urologist

☐ Clin oncologist

☐ Staff grade/ Middle grade oncologist

☐ Nurse lead: CNS Nurse lead practitioner/ Dedicated bladder cancer CNS

☐ Surgical care practitioner (SCP)

☐ Histopathologist/ Pathologist

☐ Pharmacy lead

☐ Psychologist/ Psychiatrist

☐ Other (please specify)

10. Decision Making on adjuvant intravesical Therapy for HR-NMIBC

☐ Consultant Urologist

☐ Staff grade/ Middle grade Urologist

☐ Trainee Urologist

☐ Uro-oncology surgical fellow

☐ Med oncologist

☐ Trainee Urologist

☐ Clin oncologist

☐ Staff grade/ Middle grade oncologist

☐ Nurse lead: CNS Nurse lead practitioner/ Dedicated bladder cancer CNS

☐ Surgical care practitioner (SCP)

☐ Histopathologist/ Pathologist

☐ Pharmacy lead

☐ Psychologist/ Psychiatrist

☐ Other (please specify)

11. Instillation of adjuvant Intravesical Therapy for HR-NMIBC

☐ Consultant Urologist

☐ Staff grade/ Middle grade Urologist

☐ Trainee Urologist

☐ Uro-oncology surgical fellow

☐ Med oncologist

☐ Trainee Urologist

☐ Clin oncologist

☐ Staff grade/ Middle grade oncologist

☐ Nurse lead: CNS Nurse lead practitioner/ Dedicated bladder cancer CNS

☐ Surgical care practitioner (SCP)

☐ Histopathologist/ Pathologist

☐ Pharmacy lead

☐ Psychologist/ Psychiatrist

☐ Other (please specify)

12. HR-NMIBC patient surveillance/ follow ups (monitoring for recurrence/progression)

☐ Consultant Urologist

☐ Staff grade/ Middle grade Urologist

☐ Trainee Urologist

☐ Uro-oncology surgical fellow

☐ Med oncologist

☐ Trainee Urologist

☐ Clin oncologist

☐ Staff grade/ Middle grade oncologist

☐ Nurse lead: CNS Nurse lead practitioner/ Dedicated bladder cancer CNS

☐ Surgical care practitioner (SCP)

☐ Histopathologist/ Pathologist

☐ Pharmacy lead

☐ Psychologist/ Psychiatrist

☐ Other (please specify)

13. TURBT to remove recurrent NMIBC

☐ Consultant Urologist

☐ Staff grade/ Middle grade Urologist

☐ Trainee Urologist

☐ Uro-oncology surgical fellow

☐ Med oncologist

☐ Trainee Urologist

☐ Clin oncologist

☐ Staff grade/ Middle grade oncologist

☐ Nurse lead: CNS Nurse lead practitioner/ Dedicated bladder cancer CNS

☐ Surgical care practitioner (SCP)

☐ Histopathologist/ Pathologist

☐ Pharmacy lead

☐ Psychologist/ Psychiatrist

☐ Other (please specify)

14. Bladder Sparing Treatment options at NMIBC recurrence/progression

☐ Consultant Urologist

☐ Staff grade/ Middle grade Urologist

☐ Trainee Urologist

☐ Uro-oncology surgical fellow

☐ Med oncologist

☐ Trainee Urologist

☐ Clin oncologist

☐ Staff grade/ Middle grade oncologist

☐ Nurse lead: CNS Nurse lead practitioner/ Dedicated bladder cancer CNS

☐ Surgical care practitioner (SCP)

☐ Histopathologist/ Pathologist

☐ Pharmacy lead

☐ Psychologist/ Psychiatrist

☐ Other (please specify)

15. Identification of suitable HR-NMIBC candidates for Clinical trials (newly diagnosed or recurrent/progressive)

☐ Consultant Urologist

☐ Staff grade/ Middle grade Urologist

☐ Trainee Urologist

☐ Uro-oncology surgical fellow

☐ Med oncologist

☐ Trainee Urologist

☐ Clin oncologist

☐ Staff grade/ Middle grade oncologist

☐ Nurse lead: CNS Nurse lead practitioner/ Dedicated bladder cancer CNS

☐ Surgical care practitioner (SCP)

☐ Histopathologist/ Pathologist

☐ Pharmacy lead

☐ Psychologist/ Psychiatrist

☐ Other (please specify)

**Section 3: High-risk non-muscle invasive bladder cancer (HR-NMIBC) diagnostic investigations**

**6A. How do new bladder cancer referrals most typically enter the management pathway in your experience?** (select all that apply)

☐ GP (General Practitioner) referral

☐ Accident and Emergency referral

☐ Via another referring NHS hospital

☐ Haematuria Clinic referral

☐ Specialist Urology Two-Week Wait (2WW) clinic referral

☐ Private setting referral

☐ Via another hospital specialty

☐ Other (please specify) ____________________________

**6B. From those selected in Question 6A, please rank the first, second and third most frequent referral route.**

**7. If you receive referrals from a Haematuria/2WW clinic at your site, who currently leads these clinics?**

☐ Dedicated Bladder Cancer CNS

☐ Advanced Nurse Practitioner

☐ Consultant Urologist

☐ Dedicated bladder cancer team

☐ Other (please specifiy):__________________________________________________________________________

**8. Does your centre adhere to the NICE criteria (*NG2*) for suspected bladder cancer referrals?**

☐ Yes

☐ No

**9. Does your local Bladder Cancer team currently have sufficient dedicated bladder cancer specialist nurse resourcing to meet foreseeable demands of HR-NMIBC patient care?**

☐ Yes

☐ No

**10. At what point in the pathway do newly diagnosed HR-NMIBC patients first get introduced to their key worker typically and what MDT members most often perform this role?**

___________________________________________________________________________________

**11A. Which of the following tools are most routinely utilized by your team at the time of diagnosis?** (Select all that apply)

☐ White light cystoscopy

☐ Photodynamic diagnosis (PDD) / Blue light cystoscopy

☐ Narrow-band imaging cystoscopy (NBI)

☐ Urine cytology

☐ CT urography

☐ PET-CT

☐ MRI

☐ Ultrasound

☐ Other (please specify) ____________________________

**11B. From those selected in Question 11A, please rank the first, second and third most utilised option.**

**12. Regarding main alternatives to White light cystoscopy, which of the following apply, in your opinion?** (select all that apply)

☐ PDD/Blue light or NBI are superior to white light as a diagnostic tool

☐ PDD/Blue light or NBI aids identification of carcinoma in situ

☐ PDD/Blue light or NBI are not superior to white light as a diagnostic tool

☐ Access to PDD/Blue light or NBI is valuable to help manage the risk of recurrence or progression of HR NMIBC

☐ PDD/Blue light or NBI may not represent a good use of NHS resource.

☐ No Access locally to PDD/Blue light or NBI

☐ Undecided

☐ None of the above

**13. Can you access a Smoking Cessation Program locally for your bladder cancer patients?**

☐ yes

☐ no

**14. At your site, what proportion of new localised bladder cancer patients are diagnosed with:**

Non-Muscle Invasive Bladder Cancer (NMIBC) _____/100

Muscle Invasive Bladder Cancer (MIBC) _____/100

Unknown/Unsure __/100

**15. In your experience, what % of newly diagnosed bladder cancer patients have incomplete or missing TNM stage? (eg pTa and pTis)**

☐ None

☐ <5%

☐ ≥5% <10%

☐ ≥10% <20%

☐ ≥20% <33%

☐ ≥33%

☐ Don’t know

**16. In your experience, what could be the main reasons why some bladder cancer patients might have unknown or missing TNM stage? (eg pTa and pTis)**

**17. In your local experience, for new bladder cancer referrals, how many days does it typically take for:**

**Initial appointment with Urologist:**

☐ Within1 week

☐ Within 2 weeks

☐ Over 2 weeks

**Initial Diagnostic Flexible Cystoscopy**:

☐ Within1 week

☐ Within 2 weeks

☐ Within 3 weeks

☐ Over 3 weeks

**Transurethral biopsy**:

☐ Within1 week

☐ Within 2 weeks

☐ Within 3 weeks

☐ Over 3 weeks

**Pathology review/ staging:**

☐ Within1 week

☐ Within 2 weeks

☐ Over 2 weeks

**Diagnosis communication with patient:**

☐ Within1 week of pathology report

☐ Within 2 weeks of pathology report availability

**Typically, at your site, overall, how long is the typical timeframe from referral to a confirmed and communicated diagnosis of HR-NMIBC?**

☐ Up to 4 weeks.

☐ Up to 6 weeks.

☐ Up to 8 weeks.

☐ More than 8 weeks.

**Section 4: NMIBC Treatment guidelines**

**18. 2015 NICE (NG2) Bladder cancer: diagnosis and management guidelines: Which aspects are most relevant to your current routine management of bladder cancer patients?** (select all that apply)

☐ Information and support for people with bladder cancer

☐ Diagnosing and staging bladder cancer

☐ Treating NMIBC

☐ Follow-up after treatment for NMIBC

☐ Treating MIBC

☐ Follow-up after treatment for MIBC

☐ Managing locally advanced or metastatic urothelial carcinoma

☐ Specialist palliative care for people with incurable bladder cancer

☐ I do not follow any of the current NICE Bladder Cancer Guidelines (2015)

**19A. Overall, which of the following guidelines/recommendations do you find most useful to inform current management of HR NMIBC?** (select all that apply)

☐ NICE (National Institute for Health and Care Excellence) Bladder cancer: diagnosis and management guidelines (2015)

☐ EAU guidelines (European Association of Urology)

☐ ESMO guidelines (European Society for Medical Oncology)

☐ GIRFT (Getting it Right First Time) Urology Guidance for Bladder Cancer

☐ AUA guidelines (American Urological Association)

☐ EORTC guidelines (European Organisation for Research and Treatment of Cancer)

☐ IBCG guidelines (International Bladder Cancer Group)

☐ NCCN^®^ guidelines (National Comprehensive Cancer Network^®^)

☐ Local/Regional guidelines

☐ Other guidelines (Please specify): ______________________________________________

**19B. From those selected in Question 19A, please rank the first, second and third most useful guidelines/recommendations**

**Section 5: High-risk NMIBC Treatment Options**

**20. At your centre how many NMIBC patients overall are typically discussed per month at MDT meetings?**

☐ <10

☐ 10-15

☐ 15-20

☐ 20-25

☐ 25-30

☐ >30

**21. Of these NMIBC cases, what proportion tend to present with CIS (carcinoma in situ) at your site?**

☐ < 10%

☐ ≥10% ≤ 15%

☐ > 15%

**22. At your centre, what proportions of NMIBC patients are risk stratified into low-, intermediate- and high-risk subgroups?**

- Low Risk _____/100
- Intermediate Risk _____/100
- High Risk (HR)_____/100
- No Risk Stratification recorded______/100

**23A. What are the main challenges in risk stratification in NMIBC patients in the real-world clinical setting?** (select all that apply)

☐ Tumour size information missing

☐ Lesion count information missing

☐ Tumour Grade missing

☐ Variability in interpretation

☐ Tumour Stage missing

☐ Recurrence history information missing

☐ Other (please specify) ____________________________

**23B. From those selected in Question 23A, please rank the first, second and third most important challenges**

**24. For patients with non-muscle invasive bladder cancer (NMIBC), how do you currently assess patient frailty?​**

(select all that apply)

☐ Comprehensive Geriatric Assessment (CGA)

☐ Fried Phenotype

☐ Modified Frailty Index (mFI)

☐ Frailty Discriminant Score (FDS)

☐ Clinical Frailty Scale (CFS)

☐ Electronic Frailty Index (eFI)

☐ Clinical opinion

☐ Rockwood Frailty Scale

☐ None

☐ Other (please specify) ____________________________

**25. At your site, do you have access to any support from a perioperative optimisation medical team?**

☐ yes

☐ no

**26A. At your centre, following Transurethral resection of bladder tumour (TURBT) for HR-NMIBC, which of the following are routinely chosen as adjuvant therapy** (select all that apply)

☐ BCG induction and 1–3 yr of maintenance

☐ Sequential Intravesical Gemcitabine & Docetaxel (Gem/Doce)

☐ Intravesical Mitomycin C (MMC)

☐ Hyperthermic Intravesical Chemotherapy (e.g. HIVEC™ or Synergo®) with mitomycin-C (MMC)

☐ Sequential BCG and electromotive MMC

☐ Intravesical Gemcitabine

☐ Clinical trial

**26B. From those selected in Question 26A, please rank the first, second and third most frequently chosen option**

**27. In your experience, for those HR-NMIBC who receive intravesical BCG what proportion typically complete:**

- Complete BCG induction followed by 2 or more years of maintenance _________________%
- Complete BCG induction followed by up to 1 year of maintenance_______________%
- Receive ≥ 5 doses of BCG induction and ≥ 2 doses of maintenance__________%
- Complete at least 5 doses of BCG induction only__________%
- Fail to complete BCG induction (<5 doses, due to disease recurrence or unacceptable toxicity/QOL impacts) ___________%

**28A. In your experience, what tend to be the most important real-world challenges when offering BCG to HR-NMIBC patients in the NHS** (Select all that apply)

☐ Waiting times for new patient BCG initiations

☐ Recurrence on/after BCG

☐ Lack of options after BCG

☐ Patient experience of BCG toxicities (eg cystitis, dysuria, polyuria, haematuria)

☐ NHS workload associated with the management of BCG Toxicities

☐ Patient experience associated hospital visits/treatment schedule/schema

☐ Patient challenges associated travel for hospital visits/treatment schedule/schema (eg rural/ location/public transport issues)

☐ Patient challenges associated work/family commitments and hospital visits/treatment schedule/schema

☐ NHS Capacity considerations associated with BCG treatment schedule/schema

☐ Patient education

☐ Patient access to psychological support

☐ Staff education

☐ BCG Shortages

☐ Other (please specify)______________________________

**28B. From those selected in Question 28A, please rank in order of importance with 1 being the most important challenge**

**29. At your centre, for BCG unresponsive* High Grade NMIBC what proportion of patients tend to be:**

**(*persistent/recurrent disease shortly after a full course of BCG, often ≤ 6 months for T1/Ta high-grade or ≤ 12 months for CIS)**

Eligible and Consent to Radical Cystectomy___________%

Eligible but Refuse Radical Cystectomy___________%

Ineligible for Radical Cystectomy___________%

**30A. At your centre, for BCG unresponsive NMIBC patients, what tend to be the most common reasons for refusing radical cystectomy (RC) despite being eligible?** (select all that apply)

☐ Patient concerns associated with Safety/Morbidity/Mortality

☐ Patient preference for bladder preservation

☐ Patient preference for preservation of sexual function

☐ Cultural aspects/beliefs

☐ Health literacy

☐ Patient QOL concerns

☐ Other (please specify)_____________________________________________________________________________________

**30B. From those selected in Question 30A, please rank the first, second and third most frequent concern**

**31A. At your centre, for BCG unresponsive NMIBC patients, which of the following bladder sparing treatment options tend to be considered most appropriate?** (select all that apply)

☐ Further intravesical BCG

☐ Intravesical Mitomycin C (MMC)

☐ Hyperthermic Intravesical Chemotherapy (e.g. HIVEC™ or Synergo®) with mitomycin-C (MMC)

☐ Intravesical Gemcitabine

☐ Sequential Intravesical Gemcitabine & Docetaxel (Gem/Doce)

☐ Sequential BCG and electromotive MMC

☐ Other (please specify)_____________________________________________________________________________________

**31B. From those selected in Question 31A, please rank the first, second and third most frequently chosen options**

**32. At present, for BCG unresponsive NMIBC patients, which (if any) of the following** **bladder sparing Clinical Trial options can your patients be considered for within your network?** (select all that apply)

☐ BCG in novel combinations

☐ Immune Checkpoint Inhibitor monotherapy

☐ Immune Checkpoint Inhibitor in novel combinations

☐ Novel Gene Therapy

☐ Selective oncolytic immunotherapy

☐ Alternative Intravesical treatment options

☐ There are no bladder sparing Clinical Trial options currently recruiting BCG unresponsive NMIBC patients locally

☐ Other (please specify)_____________________________________________________________________________________

**33A. In your experience, when offering bladder sparing care to BCG unresponsive NMIBC patients, which of the following tend to be the most important concerns** (select all that apply):

☐ Lack of access to new, evidence-based bladder sparing treatment options

☐ Surveillance for recurrence is resource-intensive for the NHS

☐ Coordinating MDT member expertise effectively can be challenging in a busy NHS clinical environment.

☐ Psychological support of patients to help manage any anxiety and facilitate consultation on treatment decisions.

☐ Practical patient challenges associated with where patients live and where clinics are located.

☐ Risk of recurrence

☐ Risk of progression to MIBC

☐ Patient frailty/comorbidities

☐ Need for Further Research: Access to clinical trials

☐ Other (please specify)_____________________________________________________________________________________

**33B. From those selected in 33A, please rank in order of importance with 1 being the most important concern**

**34. In your opinion does adhering to Benchmarks for Quality Performance Indicators (QPIs) reduce the risk of recurrence and progression while also reducing nationwide variance in NMIBC patient care?**

☐ yes

☐ no

**35. In your opinion, what are the most important areas to audit or QPIs to help identify or drive potential improvements in your bladder cancer service moving forward?**

_________________________________________________________________________________________________________________________________________________

______________________________________________________________________________________________________________

**36. Do you think that the clinical community in the UK would benefit from a National Bladder Cancer Audit?**

☐ yes

☐ no

**37. Within the High-Risk NMIBC treatment pathway, what do you think will be the most important unmet need to address in the next 12 – 18 months?**

__________________________________________________________________________________________________

**Section 6: Additional optional questions**

**38. What additional support would be helpful for you as a healthcare professional (HCP) in regard to managing and/or following up patients on new therapies?** (select all that apply)

☐ Cancer Tracker

☐ Allied Healthcare Professional educational support

☐ Specialist Nurse educational support

☐ Expert specialist HCP led Dedicated Bladder Cancer educational support

☐ HCP adverse event management app

☐ HCP internet portal

☐ Home care support

☐ Electronic/Digital HCP educational support

☐ Paper HCP educational support

☐ None needed

☐ Other (please specify) _________________

**39. In your opinion, what additional support would be helpful for patients in regard to managing and/or following up patients on new therapies?** (select all that apply)

☐ Electronic/Digital patient education

☐ Patient app

☐ Patient internet portal

☐ Home care support

☐ Paper patient education

☐ QR codes on medication packaging

☐ None needed

☐ Other (please specify) _________________

**Section 7: Mandatory**

**Have any Adverse Events have been reported during the interview? (SPAN-UK is listed in RRAMS: PCSONCA0869)**

☐ **Yes**

☐ **No**

**If yes, please state how many?** ____________________
